# Mercury Isotope Variability in Pyrenean Lake Sediments during the Late Holocene: Sources, Deposition, and Environmental Controls

**DOI:** 10.1021/acsearthspacechem.4c00402

**Published:** 2025-05-13

**Authors:** Bastien Duval, Juan Pablo Corella, Maxime Enrico, Alfonso Saiz-Lopez, Carlos A. Cuevas, Jose A. Adame, Rocío Millán, Maria J. Sierra, Sylvain Bérail, Blas L. Valero-Garcés, Alberto de Diego, Mario Morellón, Javier Rodríguez-Alonso, David Amouroux

**Affiliations:** † Universite de Pau et des Pays de l’Adour/E2S UPPA, CNRS, Institut des Sciences Analytiques et de Physico-Chimie pour l’Environnement et les Materiaux, UMR5254, Helioparc, 64053 Pau, France; ‡ Kimika Analitikoa Saila, Universidad del País Vasco/Euskal Herriko Unibertsitatea UPV/EHU, Sarriena Auzoa z/g, 48940 Leioa, Spain; § National Museum of Natural Sciences-CSIC, Serrano 115 bis, 28006 Madrid, Spain; ∥ Department of Atmospheric Chemistry and Climate, Institute of Physical Chemistry Blas Cabrera, CSIC, Serrano 119, 28006 Madrid, Spain; ⊥ Atmospheric Sounding Station, El Arenosillo, National Institute for Aerospace Technology (INTA), Mazagón, 7, Huelva, 21230 Mazagon, Spain; # CIEMAT, Department of the Environment (DMA), Avenida Complutense 40, E-28040 Madrid, Spain; ∇ Pyrenean Institute of Ecology, CSIC, Avda Montañana 1005, 50059 Zaragoza, Spain; ○ Departament of Geodynamics, Stratigraphy and Paleontology, Faculty of Geological Sciences, Complutense University of Madrid, Calle José Antonio Nováis 12, 28040 Madrid, Spain

**Keywords:** mercury, isotopes, lake sediments, legacy pollution, Pyrenees

## Abstract

Atmospheric mercury (Hg) emissions represent a persistent global threat to ecosystems and human health. Stable Hg isotopes have emerged as powerful tools to trace historical pollution sources and reconstruct depositional pathways in natural archives. In this study, we present a 4000-year reconstruction of Hg isotopic composition from two Pyrenean lake sediment records (Lake Marboré and Lake Estanya) located along an altitudinal gradient and compare them with those of a nearby ombrotrophic peatland (Estibere mire). Both lakes exhibit a long-term increase in Hg accumulation rates and shifts in isotope values since the onset of the Modern Period (∼16th century), consistent with intensified anthropogenic emissions. However, the isotopic patterns differ: Lake Estanya, located in a lowland area with historical land-use changes, reflects a more localized Hg signal, whereas the high-elevation, remote Lake Marboré preserves a broader regional atmospheric imprint, dominated by wet deposition. The comparison with Estibere mire—pristine and situated within the same air mass trajectory as Marboré—reveals a consistent offset in Δ^199^Hg values yet strikingly similar temporal trends, indicating a shared regional source signal modulated by ecosystem-specific processes. This multiarchive and multialtitude framework provides a rare opportunity to disentangle Hg source signatures from depositional and postdepositional transformations. Moreover, variations in even-MIF (Δ^200^Hg) in the alpine lake show the potential to reflect past climate phases, highlighting the additional value of Hg isotopes as paleoclimatic proxies. Our results underscore the importance of integrating different ecosystem archives to improve reconstructions of atmospheric Hg dynamics and to refine interpretations of legacy pollution and climate interactions.

## Graphical abstract

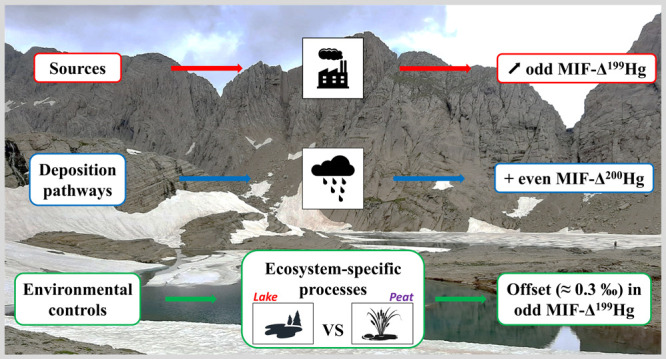

## 1 Introduction

Mercury (Hg) is a global pollutant present in all surface compartments of the Earth that affects human and ecosystem health.^1–7^ Primary anthropogenic Hg emissions greatly exceed natural sources,^7–10^ increasing Hg in reservoirs during the last millennia. The global annual mean lifetime of Hg(0) against the net photochemical oxidation is in the range of several months to over a year,^11^ and recent findings on atmospheric Hg reduction processes have postulated that global atmospheric Hg lifetime could increase by a factor of 2.^12,13^ Natural primary emission of Hg from geogenic sources consists of the release of Hg from the continental crust through natural weathering, hydrothermal activities, and volcanic degassing. Anthropogenic emissions include the use of fossil fuels (mainly coal burning), artisanal and small-scale gold mining, iron and nonferrous metals production, cement production, oil refining, and wastes from consumer products.^4–7,14^

Mercury concentration records derived from natural archives such as lake sediments,^15–17^ marine sediments,^18,19^ peat,^20–22^ and ice cores^23–26^ have already highlighted the influence of anthropogenic activities on the Hg fluxes to the environment during the last millennia. In the absence of Hg anthropogenic emissions, natural archives have also revealed the significant influence of climate variability on Hg concentration and fluxes in the Arctic,^26^ Antarctic,^27^ and tropical^28^ regions during the Last Glacial Period and the Early and Mid-Holocene (ca. 25,000 to 4000 yrs Before Present (BP)). During the last two millennia, a pioneering study in peat records from NW Spain allowed the first paleoclimatic reconstruction using accumulated Hg as a paleotemperature proxy.^21^ Unfortunately, disentangling the contribution of climate and human activities on the reconstructed Hg fluxes to the environment during the last centuries, with a significant human imprint on the Hg cycle, remains a scientific challenge.

Mercury isotopes have provided new insights into Hg cycling processes during the past decade, contributing to deciphering the natural (e.g., climate) vs human influence driving mercury fluxes to the Earth’s surface.^29–32^ Mercury has seven stable isotopes that undergo mass-dependent fractionation (MDF, δ^202^·Hg) during both kinetic and equilibrium reactions as a result of many physical, chemical, or biological processes such as evaporation, complexation or binding to ligands, microbial reduction and methylation/demethylation, photochemical reactions, and some metabolic processes in living organisms involving Hg compounds transformation such as in vivo demethylation.^29,33^ Mass-independent fractionation (MIF) has now been well established for both odd and even Hg stable isotopes, especially during light-induced reactions. Odd-MIF of Hg isotopes (Δ^199^Hg, Δ^201^Hg) is primarily related to photochemical reactions such as photoreduction of Hg­(II) and photodegradation of MeHg.^34^ Positive anomalies in Δ^200^Hg are generally attributed to photochemical oxidation of elemental mercury (Hg^0^) occurring in the upper atmosphere, near the tropopause, although the exact mechanisms remain unclear.^35–38^ This is particularly relevant given the significant fraction of Hg that has been recently demonstrated to be chemically processed in the lower stratosphere.^39^ Δ^200^Hg was further proposed as a proxy for the respective contribution of dry and wet atmospheric deposition of Hg.^40^ In natural archives, most Hg isotopic analyses have been carried out in lakes and peatlands to evaluate historical variations of Hg isotopic fingerprints.^17,22,36,41–50^ Enrico et al.^22,36^ used the distinct, conservative even-MIF signatures of rainfall and atmospheric gaseous Hg(0) to discriminate the main deposition pathways in two remote peatlands and reconstructed past atmospheric Hg levels. Dry deposition, characterized by slightly negative Δ^200^Hg,^36,51^ involves foliar uptake of Hg(0),^52^ whereas wet deposition, with positive Δ^200^Hg,^35,36^ involves the scavenging of gas-phase and aerosol-phase Hg­(II) by cloud droplets. In the Northern Hemisphere, wet deposition is characterized by significant positive even-MIF Δ^200^Hg^22,36,41,53,54^ whereas GEM dry deposition (Hg(0)) shows slight negative Δ^200^Hg.^36,41,51,53^ For MDF and odd-MIF changes in isotopic values, a widely observed significant change in the Hg isotopic signatures from sedimentary archives, with increasing δ^202^Hg and Δ^199^Hg variability, occurs during periods corresponding to the increased Hg emissions related to local and/or regional industrial development.^22,41,43,46,47,49,55^

The Iberian Peninsula is a global hotspot of mercury pollution with a long history of Hg mining activities, including the world’s largest Hg mines located in southern Spain (Almadén mining district) (Figure 1A). Almadén mines have alone contributed approximately one-third of the globally mined Hg, with an accumulated historic Hg emission estimate of 10,000 tons.^14^ Anthropogenic Hg pollution due to Hg mining activities in Almadén ca. 2500 yrs BP was documented in peat archives from NW Spain^21^ and in Posidonia oceanica mats along the northwest Mediterranean coast.^18^ Furthermore, Hg ancient mining in sulfide ore deposits from Southern Spain, exploited during the Copper Age, has resulted in the World′s earliest evidence of mercury pollution recorded in Río Tinto estuary floodplain sediments (SW Spain) at the onset of the Late Holocene (ca. 4500 yrs Before Present (BP).^56^ Surprisingly, there is a lack of Hg isotopic studies from natural archives in the Iberian Peninsula to understand Hg legacy pollution sources and Hg biogeochemical cycling in the cradle of humankind’s Hg production.

**Figure 1:**
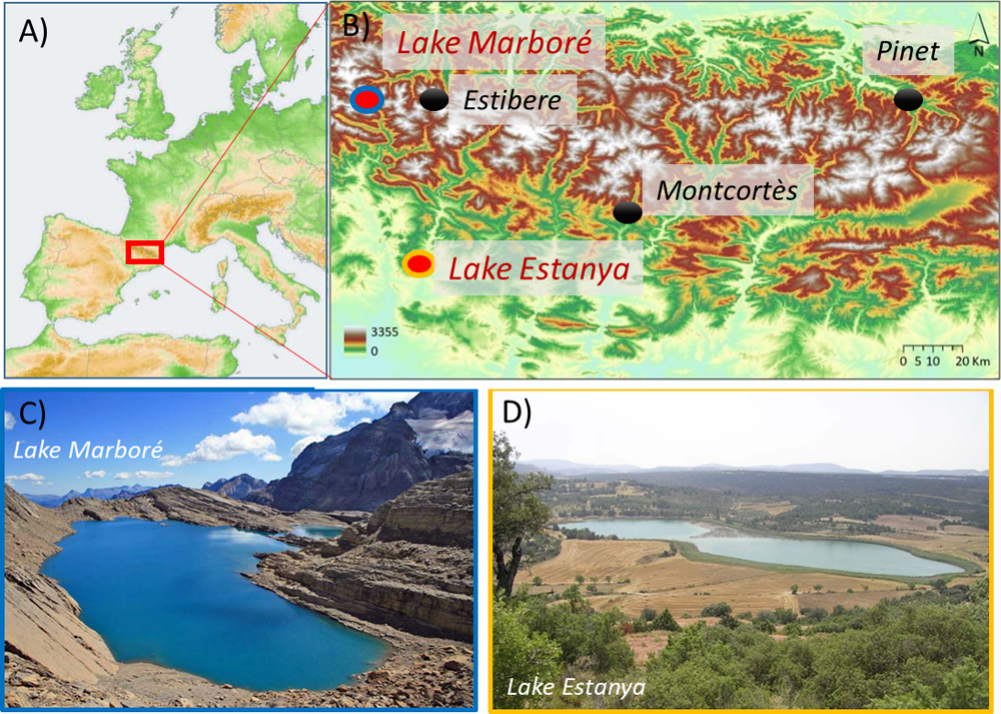
Location of the study sites; (A) Map of Europe; (B) Digital Elevation Model for the Central and Eastern Pyrenees showing the location of Lakes Estanya (42°02′N; 0°32′E, 670 m asl) and Marboré (42°41′N; 0°2′E, 2612 m asl) and other Hg records mentioned in the text; (C, D) Photographs of the lakes and their watersheds.

In this study, we selected two lacustrine sedimentary records from the Southern Central Pyrenees, located along an altitudinal gradient (Figure 1), to investigate the long-term variability of mercury isotopic composition during the Late Holocene. Our specific aims were: (i) to assess how site-specific environmental factors (e.g., climate, geomorphology, and land use) influence Hg accumulation in different lake systems; (ii) to compare the Hg isotopic signatures from lake sediments with those from a nearby peat record to evaluate how depositional environments and ecosystem types shape the isotopic imprint of atmospheric Hg; and (iii) to explore the potential of Hg stable isotopes as proxies to disentangle natural (climatic) and anthropogenic drivers of Hg deposition in southwestern Europe. We also consider that historical changes in catchment disturbance and vegetation cover—linked to deforestation and land-use change over the last centuries—may have influenced the dominant Hg deposition pathways (e.g., enhanced dry deposition in more vegetated settings) and thus contributed to the isotopic variability observed in the sedimentary records. However, we hypothesize that remote high-altitude sites, such as peatbog Estibere and the alpine lake Marboré investigated here, have remained largely unaffected by recent catchment alterations and therefore preserve atmospheric Hg signals representative of broader regional trends. We further hypothesize that differences in Hg accumulation and isotopic signals are primarily driven by altitudinal and ecological contrasts between lake catchments; that despite depositional differences, lakes and peatlands may preserve common regional pollution trends; and that variations in both odd- and even-MIF reflect shifts in climate and anthropogenic Hg emissions, offering new opportunities for paleoclimatic and environmental reconstructions.

## 2 Material and Methods

### 2.1 Study Site

The two studied lacustrine ecosystems, Lake Marboré (42°41′N; 0°2′E, 2612 m asl) (Figure 1C) and Lake Estanya (42°02′N; 0°32′E, 670 m asl) (Figure 1D), are located in the Southern Central Pyrenees. They show similarities with small surface lake areas of 0.143 and 0.188 km^2^, small-sized watersheds of 13.7 and 10.6 km^2^, and maximum depths of 30 and 24 m in lakes Marboré and Estanya, respectively.^57,58^ The watersheds of both lakes are emplaced over carbonate bedrocks.^59–61^ Bioclimatic conditions in both lakes greatly differ with lower temperatures and higher precipitation in Lake Marboré (mean annual temperatures and precipitation of 5 °C and 2000 mm) compared to Lake Estanya (mean annual temperatures and precipitation of 14 °C and 470 mm).^58,59^

Lake Marboré is a high-alpine lake located above the tree line. The lake’s hydrology is controlled by precipitation/evaporation balance, meltwater input along a small NW inlet, outputs through a surface outlet located in the southern area, and some groundwater fluxes.^58,62^ It is a cold dimictic and ultraoligotrophic lake with alkaline waters. Ice and snow cover Lake Marboré surface 9–10 months per year.^63^

Lake Estanya is a karstic lake emplaced in Triassic carbonate, marls, and claystones.^64^ It is a monomictic lake with brackish and oligotrophic waters.^64^ Vegetation in the watershed consists of scrublands and oak forests in the high-elevated areas, while the lowlands are mostly covered by barley cultivation.^64^

The contrasting land-use histories of the two lake catchments are crucial for interpreting the Hg isotopic signals preserved in the sediments. Lake Marboré, due to its high elevation and location above the treeline, has remained largely unaffected by direct human activities throughout the Holocene. Its watershed is dominated by bare rock and sparse alpine vegetation, with no historical records of deforestation or land use.^65,66^ In contrast, Lake Estanya has experienced significant anthropogenic disturbance, particularly since the Medieval period.^64^ Historical and paleoenvironmental records indicate widespread deforestation and the expansion of agricultural activities in its watershed, which intensified erosion and sediment input into the lake.^64,67^

### 2.2 Air Masses Back-Trajectories Analyses

Back trajectories with the arrival point to the studied lakes were investigated to explore the mercury atmospheric transport pathways during the last few decades. The HYSPLIT (Hybrid Single-Particle Lagrangian Integrated Trajectory) model^68^ was used to establish source–receptor relationships.^69^ The calculation method combined Lagrangian and Eulerian approaches using (i) a moving frame of reference for advection and diffusion calculations as the trajectories on air parcels move from their origin and (ii) a fixed three-dimensional grid as a frame of reference to compute pollutant air concentrations.^69^ The three-dimensional kinematic trajectories were computed daily at 12:00 UTC, at 100 m above ground level, and at a 72-h runtime. The meteorological fields from the global meteorological model from the European Centre for Medium Range Weather Forecasts were used and converted to the standard format. ERA40 data reanalysis was used for the computation of back trajectories in the period 1960–1993 yrs CE, while ERA-Interim was used for the period 1994–2016 yrs CE.^70^ These fields have a 0.5° spatial resolution, 22 pressure levels from the surface to 250 mb, and 6 h of temporal resolution. A frequency map with the number of back-trajectories passing above every location before arriving at the lakes was generated (Figure S1 and supplementary text summarize the recent air masses back-trajectories in the Central Pyrenees). To quantify the main source areas of these trajectories, several regions were defined, and the number of trajectories passing through each region before arriving at the study sites was calculated (back trajectories percentages passing over different regions and arriving at Lake Estanya and Lake Marboré are summarized in Table S1).

### 2.3 Sediment Sequence and Age-Depth Models

Both lake sequences have been intensively studied from sedimentological, geochemical, and palynological points of view^67^ and have robust ^14^C, ^210^Pb, and ^137^Cs–based age models. Sediment cores were retrieved from the deepest area of the studied lakes using a UWITEC (MAR11-1U sediment core, 27 m depth, 2011)^61^ and Kullemberg (LEG04-1K sediment core, 24 m depth, 2004)^64^ floating platforms for Lake Marboré and Lake Estanya, respectively. UWITEC gravity cores were additionally collected to preserve the uppermost sediments and the water-sediment interphase in both lakes (MAR11-1G-1U, LEG1A-1 M sediment cores).^57,61^

The age-depth models in the two lakes are based on ^137^Cs, ^210^Pb, and Accelerator Mass Spectrometry (AMS) ^14^C radiometric dating techniques. The Holocene chronology for Lake Marboré and Lake Estanya sediment sequences was developed using 10 and 11 AMS ^14^C dates, respectively. ^210^Pb and ^137^Cs radiometric dating was applied to the recent sediment in both lakes. The mean annual sedimentation rate (SR) in Lake Marboré was constant during the Late Holocene (SR ≈ 0.6 mm yr^–1^) while SR in Lake Estanya ranged from 0.2 to 2.1 mm yr^–1^.^57,61,64,66^

The selected section for Lake Marboré spans the last 3 ka, and it is composed of laminated to banded fine silts composed of silicate minerals and very low organic and carbonate content.^61^ In this lake, 27 sediment samples have been selected for Hg isotopic analyses with ∼110 years of mean chronological resolution. The Estanya section spans the last 4 ka and includes carbonate-rich silts of mainly detrital origin deposited under relatively high lake level conditions and increased runoff and organic-rich facies with gypsum formed under shallower conditions.^57,64^ In this lake, 20 sediment samples have been selected for Hg isotopic analyses with ∼200 years of mean chronological resolution for the studied period. According to the sample resolution and age-depth models from both sedimentary archives, the paleoenvironmental information provided in this study corresponds to centennial time scales during the Late Holocene. For this reason, we increased the sampling resolution during the last five centuries, achieving a mean chronological resolution of ∼50 and ∼35 years for Lake Estanya and Lake Marboré, respectively, which allowed us to explore the human imprint on the Hg isotopic signature during the Modern Period.

### 2.4 Mercury Concentrations and Fluxes

Mercury analyses were carried out in discrete samples retrieved downcore in the studied cores (see the Supporting Information for additional methodological and experimental details). Total Hg concentration measurements were carried out by pyrolysis gold amalgamation and atomic absorption spectroscopy (AAS) using an Advanced Mercury Analyzer (AMA-254, LECO Company). Certified reference material (CRM) was used to determine the accuracy and precision of the Hg measurements (NCS DC 87103, soil, [Hg] = 17 ± 3 μg kg^–1^). The repeatability was *S*_r_ ≤ 15%, and the relative uncertainty associated with the method (*k* = 2) was ± 20%. All analyses were run at least in triplicate. Mass accumulation rates for Hg depositional flux estimation (HgAR) were calculated based on the Hg concentration in the sediment, the dry bulk density of the sediment, and the sedimentation rates according to Givelet et al.^71^

### 2.5 Mercury Stable Isotopes Analyses

The analytical protocol for the determination of Hg isotopes in sediments is derived from previous studies.^43,72^ Before Hg isotopic analysis, sediment samples (0.5–1 g) were first predigested overnight at room temperature in a Teflon tube using 3 mL of nitric acid (65%, INSTRA quality). After the addition of 1 mL of hydrochloric acid (37%, INSTRA quality), the extraction of Hg was carried out using a Hotblock at 85 °C (6 h plus 3 h after the addition of about 1.3 mL of hydrogen peroxide (30%, ULTREX quality)). Then, an aliquot of about 1.5 mL was recovered in an Eppendorf Safe-Lock tube and centrifuged at 14,500 rpm for 90 s. The supernatant was collected and diluted for isotopic measurements (10% HNO_3_, 2% HCl, either 0.5 or 1 μg kg^–1^ of Hg depending on the analytical session). Hg isotopic composition was determined using a cold-vapor generator (CVG) with SnCl_2_ reduction coupled with MC-ICPMS (Nu Instruments). NIST SRM-997 thallium standard solution was used for mass-bias correction. Sample standard bracketing with NIST SRM-3133 was conducted to report Hg isotopic values as delta notation to allow interlaboratory comparisons.^73^ (Detailed analytical procedure is summarized in the Supporting Information and Tables S2 and S3).

Reference material NIST-8610 (formerly UM-Almadén) was analyzed regularly along with samples (*n* = 44) in each analytical session to validate each analytical session. The uncertainty on Hg isotope ratios is evaluated using multiple analyses of a procedural CRM (IAEA-405, estuary sediment) prepared using a procedure similar to samples. Tables S2 and S3 show the results obtained and are in good agreement with previously published values. In this article, all reported analytical uncertainties for Hg isotopic values are presented as the 2SD of IAEA-405. Sample standard bracketing with NIST SRM-3133 also allowed us to calculate a Hg recovery related to the extraction of Hg from the sediment samples.^43,74^ Recoveries averaged 103 ± 10% (*n* = 15) and 98 ± 10% (*n* = 20), respectively, for Lake Marboré and Lake Estanya.

The mass balance between wet and dry (GEM) deposition in lakes Marboré and Estanya is calculated according to Enrico et al.^22^ using the following formula [Disp-formula eq1]:

$$\Delta^{200}\mathrm{Hg}=F_{\mathrm{Wet}}\times \Delta^{200}{\mathrm{Hg}}_{\mathrm{Wet}}+F_{\mathrm{Dry}}\times \Delta^{200}{\mathrm{Hg}}_{\mathrm{Dry}}F_{\mathrm{Wet}}+F_{\mathrm{Dry}}=1$$ 1

where modern Δ^200^Hg_Wet_ end-member derived from precipitation (0.21 ± 0.04 ‰ (1σ)^36^ and Δ^200^Hg_Dry_ end-member derived from atmospheric GEM (−0.05 ± 0.04 ‰, 1σ,^36^ both parameters calculated in the Pyrenees in previous studies.^22,36^ The assumption to use this formula in our natural archives relies on the hypothesis that the Δ^200^Hg signature in wet and dry deposition did not change over time and that geogenic and anthropogenic Δ^200^Hg values are similar based on literature data.^6^

## 3 Results and Discussion

### 3.1 Evolution of Mercury Accumulation in Lacustrine Sediments

Three distinct periods of Hg accumulation can be distinguished in both the lakes Estanya and Marboré (Figure 2).

**Figure 2:**
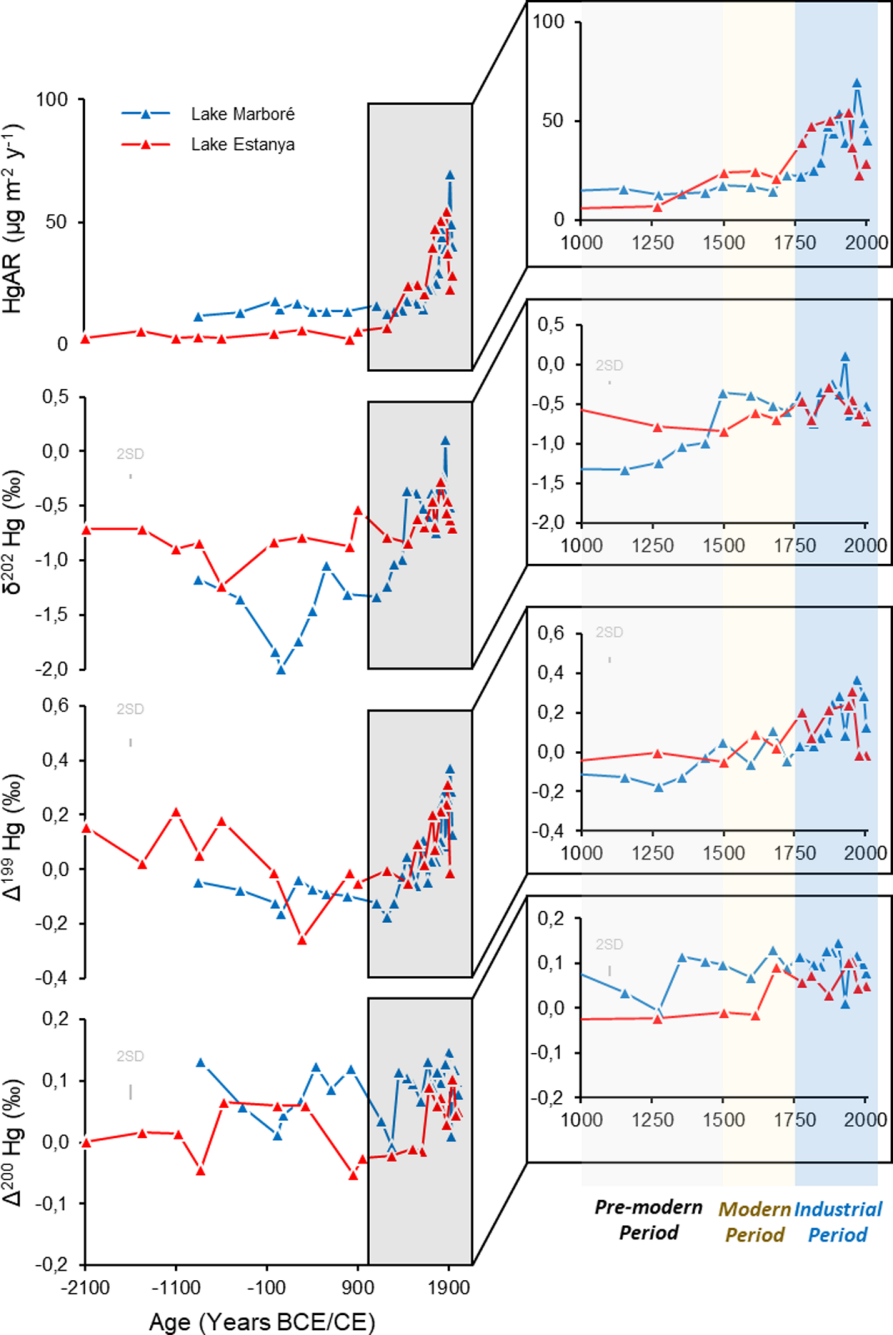
From top to bottom, Late Holocene mercury accumulation rates variability (HgAR) and Hg isotopic composition variability (δ^202^Hg, Δ^199^Hg and Δ^200^Hg) in Lake Marboré and Lake Estanya. Color bands represent the main historical periods).

#### 3.1.1 Premodern Period (2000 BCE–1500 yrs CE)

Hg accumulation rates (HgAR) were relatively low in both lakes, averaging 14.4 ± 1.7 μg m^–2^ yr^–1^ (*n* = 12) in Lake Marboré and 4.3 ± 1.7 μg m^–2^ yr^–1^ (*n* = 10) in Lake Estanya. These values are consistent with preanthropogenic Hg fluxes reported in nearby peatlands and karstic lakes from the Central Pyrenees^22,75^ (see location in Figure 1). However, it is important to note that Hg production in the Almadén mines began intermittently during the Roman period,^76^ so only the earliest part of the record—before the first millennium BCE—can be considered representative of true natural background levels. Differences in HgAR between the two lakes likely reflect site-specific environmental factors. First, Lake Marboré receives significantly higher annual precipitation due to its high-elevation setting, enhancing Hg deposition.^58,59^ Second, its watershed is composed mostly of bare rock with minimal vegetation, in contrast to Lake Estanya, which is surrounded by forest and agricultural land capable of trapping atmospherically deposited Hg through canopy uptake.^77^ Third, snow and ice cover Lake Marboré for up to 9–10 months per year,^63^ and snowpack is known to act as a reservoir for Hg that is released during spring melt.^78^ This process may promote more efficient Hg delivery to the lake bottom with minimal recycling, as observed in other alpine and Antarctic lakes.^79,80^

#### 3.1.2 Modern Period (1500–1850 CE)

This period is characterized by a progressive increase in HgAR for both Lake Marboré and Lake Estanya, with mean HgAR of 24.6 ± 10.4 μg m^–2^ y^–1^ (*n* = 8) and 23.2 ± 2.0 μg m^–2^ y^–1^ (*n* = 3), respectively. This trend corresponds to the increase in Hg production worldwide and especially in these European regions with the Almadén mines.^14^ It is worth noting that the increase in HgAR observed in Pyrenean lakes occurred earlier than the industrial rise reported in North American lakes (at around 1850s CE),^41,46^ in good agreement with the delayed increase in Hg production in North America.^14^ This difference between environmental archives from both continents from the Northern Hemisphere suggests that Hg deposited in remote lakes can be largely influenced by regional sources rather than global ones.^10,59,75^

#### 3.1.3 Industrial Period (1850 CE–Present Day)

The onset of industrialization is well observed in Lake Marboré and Lake Estanya with mean HgAR of 49.2 ± 11.7 μg m^–2^ y^–1^ (*n* = 6) and 46.0 ± 7.3 μg m^–2^ y^–1^ (*n* = 5). It is noticeable the good agreement between the modern and industrial HgAR values in both lakes, although the background levels differed considerably, suggesting a common anthropogenic driver. Although both lakes show similar HgAR trends, the low amplitude changes in the Lake Marboré record may reflect regional-scale changes as influenced by direct atmospheric inputs. On the other hand, Lake Estanya is influenced by watershed-scale human activities such as farming, deforestation, etc. Additionally, air mass back-trajectory analyses using the HYSPLIT model (Table S1, Figure S1, and Supporting Information show detailed descriptions of the recent air-mass trajectories arriving at both lakes) revealed that Lake Marboré is influenced by a greater proportion of air masses originating from distant regions, such as SW France and Central Spain, compared to Lake Estanya. This atmospheric connectivity likely enhances its capacity to integrate regional-scale Hg inputs, reinforcing its interpretation as a regional atmospheric archive.

### 3.2 Changes in Mercury Isotope Composition in Pyrenean Lacustrine Sediment Cores

Our isotopic results reveal distinct fractionation patterns in MDF, odd-MIF, and even-MIF that align with Hg accumulation rates (HgAR) observed in the sediment cores (Figure 2) (Figures S2–S4 provide Hg concentration, fluxes, and isotopic Hg results from Lake Estanya and Lake Marbore sediment cores and other sites mentioned in the text). δ^202^Hg range from −1.99 (90 CE) to 0.11‰ (1930 CE) at Lake Marboré sediments, while we observe narrower variations from −1.24 (574 BCE) to −0.28‰ (1870 CE) at Lake Estanya. The same pattern is observed for odd-MIF results with Δ^199^Hg values at Lake Marboré and Lake Estanya ranging from −0.18 (1270 CE) to 0.37‰ (1970 CE) and −0.25 (324 CE) to 0.31‰ (1950 CE), respectively. Both preindustrial background δ^202^Hg and Δ^199^Hg and their increasing trend are in agreement with the trends previously reported in lake sequences from other regions at a global scale.^40^ The profiles reveal increases in both δ^202^Hg and Δ^199^Hg in the upper part of the sediment cores corresponding to the last centuries. As expected, the even-MIF anomalies Δ^200^Hg exhibit lower variations among the samples, with values ranging from −0.01 (1270 CE) to 0.15 ‰ (1910 CE) for Lake Marboré and from −0.05 (850 CE) to 0.11 ‰ (1950 CE) for Lake Estanya sediments. At Lake Estanya, Δ^200^Hg values begin to increase significantly in the 16th century, coinciding with the onset of the Modern Period. Lake Marboré exhibits consistently higher Δ^200^Hg values than Lake Estanya throughout the record.

The Lake Estanya record δ^202^Hg and Δ^199^Hg do not display a significant difference between Premodern Period (respectively −0.82 ± 0.18 ‰ and 0.03 ± 0.14 ‰) and Modern Period (respectively −0.71 ± 0.12 ‰ and 0.02 ± 0.07 ‰) values (*t* test, *p* > 0.05), while HgAR has shown an important increase in the lake at the beginning of the 16th century (Figures 2 and 3). The smaller δ^202^Hg and Δ^199^Hg variations at Lake Estanya (except Δ^200^Hg) during this period suggest no substantial changes over this period, mainly controlled by local factors in the catchment and the lake itself, modulating Hg accumulation and isotope fractionation. Two possible site-specific lake factors might cause MDF and reduce the range of the sediment signatures: (i) a strong influence of the catchment via vegetation uptake and limnological processes (biological activity) in the lake and (ii) changes in historical land use. Indeed, a significant change in the lake level and surrounding vegetation took place in Lake Estanya during the 16th century caused by deforestation and land-use changes for agricultural activities^64,67^ that also triggered the highest runoff and soil erosion rates in the lake that could remobilize Hg stored in soils via vegetation uptake eventually deposited in the lake sediments. Lake Estanya was also strongly affected by the hydrological fluctuations that occurred in the area during the Little Ice Age (LIA) (14th to 19th centuries CE)^81,82^ resulting in abrupt lake level, organic matter, and diatom productivity oscillations that might have impacted the Hg isotopic trends. The Industrial Period was characterized by higher δ^202^Hg and Δ^199^Hg (respectively −0.49 ± 0.15 ‰ and 0.21 ± 0.09 ‰) recorded in the lake in agreement with other sedimentary records located in different bioclimatic and geographical regions worldwide^40^ that usually show a positive shift in the δ^202^Hg and Δ^199^Hg values along with the intensification of industrial activities emitting Hg to the atmosphere, such as coal power plants, cement plants, chlor-alkali plants, and waste incinerators^22,41,43,46,83^ (Figure 3).

**Figure 3:**
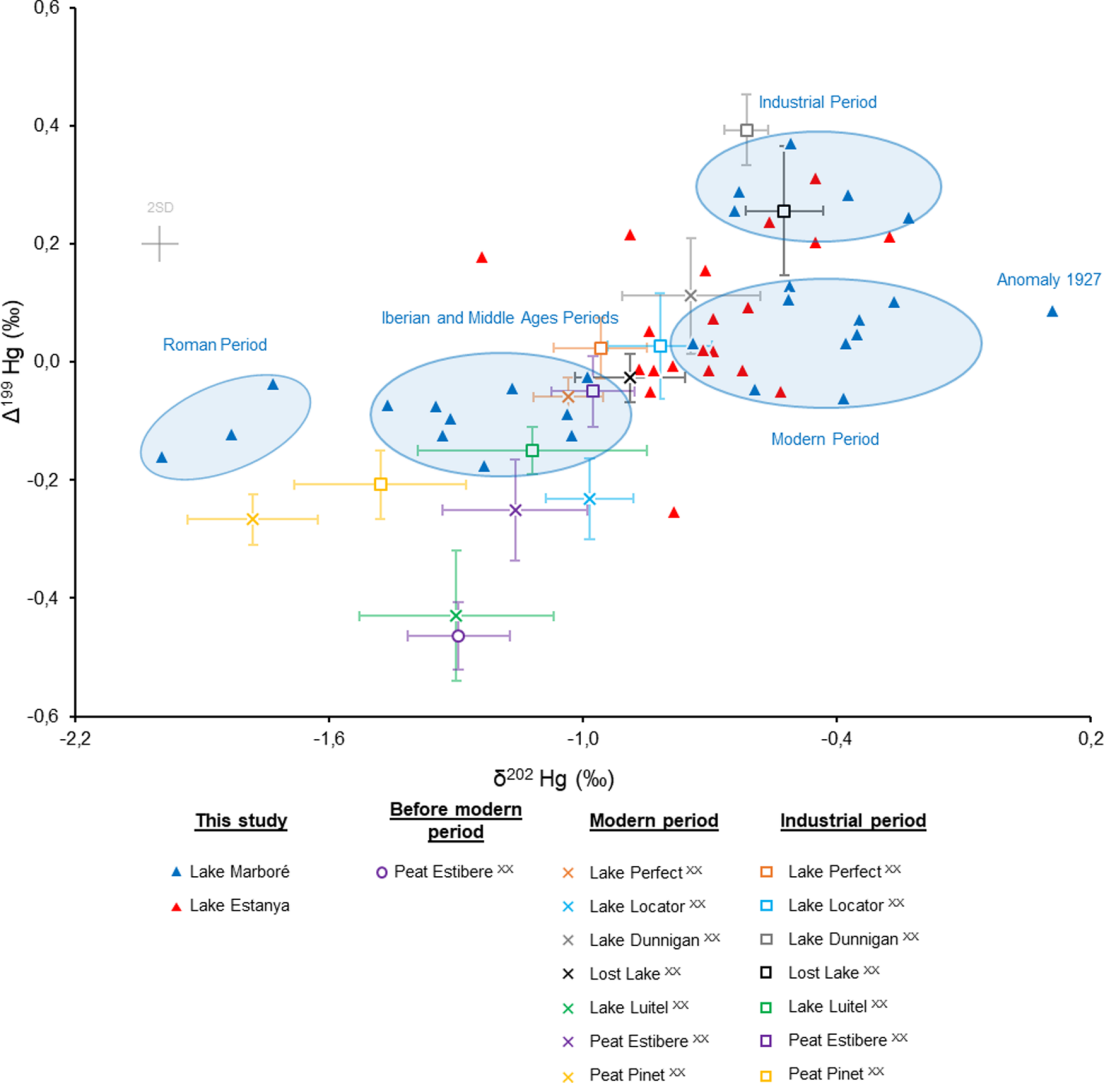
δ^202^Hg vs Δ^199^Hg plot for both Lake Marboré and Lake Estanya together with literature data: both MDF and odd-MIF increase along with contamination.

The Hg isotopic signal from Lake Marboré displays five distinct periods (Figure 4): (i) Iberian Period (840 BCE to 20 CE), (ii) Roman Period (20 to 440 CE), (iii) Middle Ages Period (440 to 1500 CE), (iv) Modern Period (1500 to 1890 CE), and (v) Industrial Period (1890 to 2000 CE). The Iberian and Middle Ages Periods, reflecting a period of limited anthropogenic influence on Hg cycling, establish a consistent baseline (δ^202^Hg = −1.21 ± 0.17 ‰ and Δ^199^Hg = −0.09 ± 0.05 ‰). This baseline condition is interrupted by an MDF of Hg isotope change during the Roman Period (δ^202^Hg = −1.85 ± 0.13 ‰ and Δ^199^Hg = −0.11 ± 0.06 ‰), expressed as a δ^202^Hg shift by −0.6 ‰. The isotopic shift toward more positive values at 280 yrs CE is coherent with the increase in Hg enrichment previously documented in the lake since the Roman era^16^ and a higher release from mining activities in Almadén mines during the last two millennia (Figures 3 and 4). Indeed, cinnabar (HgS) extraction from Almadén mines for pigment production (vermilion) is well documented and evidenced by archeological studies, in particular the numerous coins, medals, vessels, and other historical objects found in the Almadenejos and Valdeazogues areas.^76,84^ The production processes included grinding of mined cinnabar, followed by drying in furnaces. Romans collected Hg after evaporation and called it hydrargyrum (hence Hg). Arabs brought to Iberia new processes for obtaining Hg, by ore melting and sublimation. The introduction of this new technology might be responsible for some of the changes observed around the seventh–9th centuries^85^ (Figures 2–4). Depending on the minerals associated with cinnabar, quartzite, breccia, goethite, or pyrite, and depending on the location of the vein (Almadén, El Entredicho, Nuevo Entredicho, Las Cuevas or Nueva Concepción), δ^202^Hg in cinnabar varies greatly from −1.73 to 0.15 ‰.^85^ Experimental studies show that Hg isotope signatures can also be altered during ore processing: retorted Hg vapor is enriched in heavier isotopes compared to the source cinnabar,^85^ and similar enrichments have been observed during coal combustion.^86^ Hence, the decrease in δ^202^Hg recorded at Lake Marboré might relate to the interplay between the vein type exploited by Romans and the processing techniques.^85^ Given the processing methods used at Almadén, historical emissions likely consisted mainly of Hg^0^ gas, enabling long-range transport, while particulate-bound Hg would have had more localized effects. More Hg isotopic analyses, depending on the extraction process and the variety of cinnabar, are needed to better understand this negative shift during the Roman Period.

**Figure 4:**
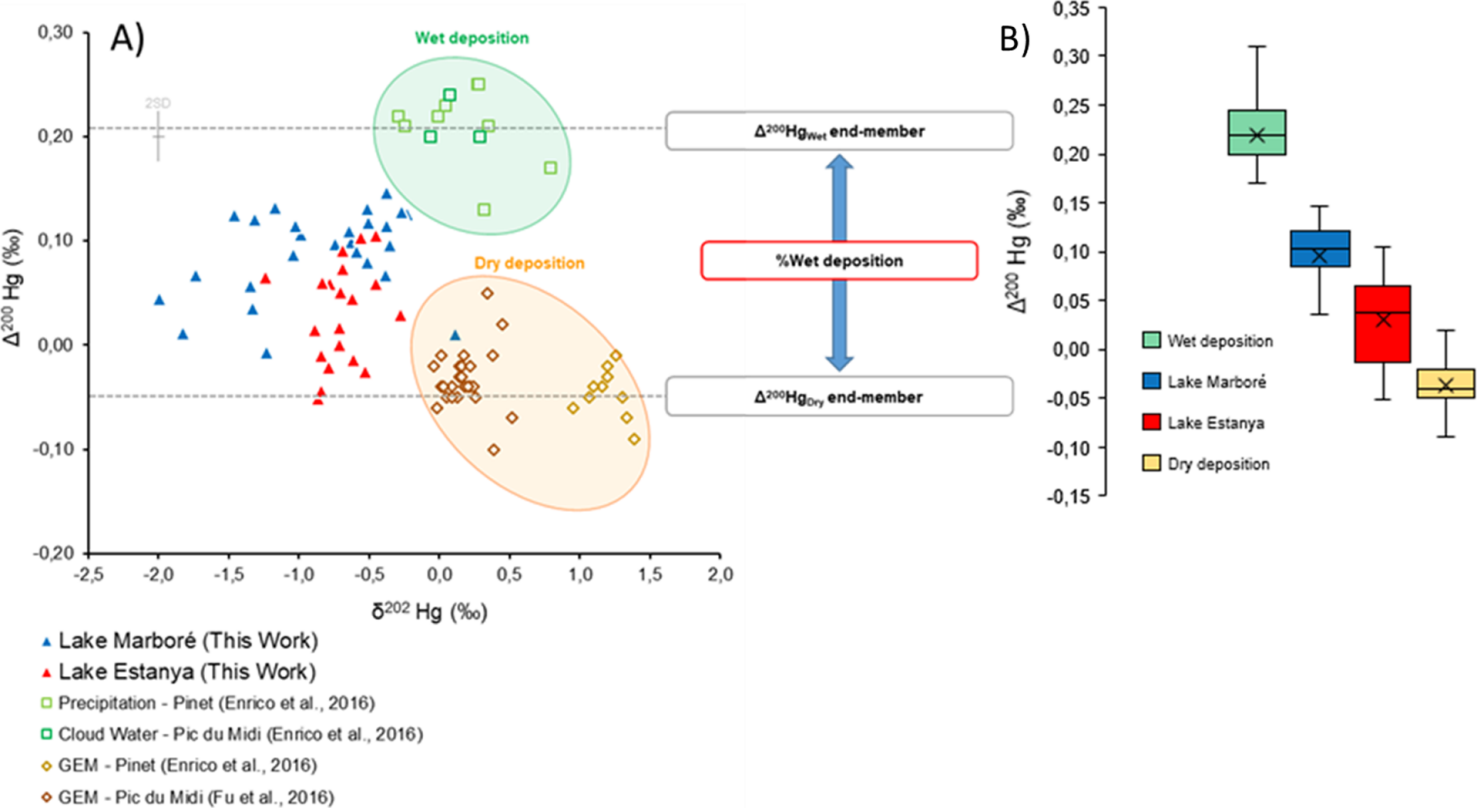
(A) δ^202^Hg vs Δ^200^Hg diagram for both Lake Marboré and Lake Estanya together with typical wet (cloud waters and precipitations) and dry (GEM) deposition Hg isotope signatures in the Central Pyrenees; (B) Δ^200^Hg box plot with the mean Lake Estanya and Lake Marbore isotopic values and wet and dry deposition isotopic ranges in the Pyrenees. Wet deposition includes data from Precipitation-Pinet and from Cloud water–Pic du Midi.^36^ Dry deposition includes data from GEM-Pinet^36^ and from GEM-Pic du Midi.^51^

The Modern and Industrial Periods are characterized by less negative δ^202^Hg values of −0.45 ± 0.16 ‰ and −0.38 ± 0.29 ‰, respectively (*t* test, *p* < 0.05), although the main Hg isotopic feature regarding Hg pollution in Lake Marboré is related to the odd-MIF signal with lower values during the Iberian and Middle Ages Periods (Δ^199^Hg = −0.09 ± 0.05 ‰), a moderate increase during the Modern Period (Δ^199^Hg = 0.04 ± 0.06 ‰), a significant increase during the Industrial Period (Δ^199^Hg = 0.26 ± 0.09 ‰), followed by a clear decrease during the last decades (Δ^199^Hg = 0.13 ‰) that follows a similar trend to HgAR (Figure 2). This earlier late-industrial decline in HgAR is also observed in recent lake sediment^41,43,46^ and peat cores^22^ and corresponds with the local to regional decline in Hg emissions due to the reduction of metal and chlor-alkali industries and decline of mining production in Europe, as well as improved technologies limiting Hg industrial emissions.^5^

A strong linear relationship between both 1/HgAR and Δ^199^Hg (*r* ^2^ = 0.72; *p* < 0.05) is observed in Lake Marboré, supporting a mixing model between two distinct end-members of atmospheric Hg inputs. This trend enables the discrimination between anthropogenic signature (near-zero Δ^199^Hg at high Hg concentrations) and a background atmospheric end-member with negative Δ^199^Hg values (likely associated with wet deposition or photochemical processing). A similar, though slightly weaker, relationship is also identified in Lake Estanya sediments from the 16th century onward (*r* ^2^ = 0.57; *p* = 0.018) (Figure S2A shows the Δ^199^Hg vs 1/HgAR plot for both Lake Marboré and Lake Estanya). These linear relationships have previously been documented in other systems such as Lost Lake (*r* ^2^ = 0.85; *p* < 0.05),^41^ Lake Luitel (peatland lake) (*r* ^2^ = 0.79; *p* < 0.05),^43^ Estibere mire (*r* ^2^ = 0.56; *p* < 0.05) and Pinet mire (*r* ^2^ = 0.50; *p* < 0.05)^22^ (Figure S2B shows the Δ^199^Hg versus 1/Hg plot for both Lake Marboré and Lake Estanya, together with other lakes and peats mentioned in the text). Interestingly, while slopes remain relatively consistent across lakes and peatlands, the *y*-intercepts vary systematically, suggesting differences in the baseline Δ^199^Hg values associated with preindustrial mercury sources. Peatlands tend to converge toward Δ^199^Hg ≈ 0‰, consistent with dominant dry deposition and limited photochemical processing, whereas lakes display elevated intercepts (+0.3 to +0.5‰), likely reflecting a greater influence of wet deposition and/or photochemical reduction within the water column. This systematic offset underscores the combined role of ecosystem-specific deposition pathways and historical source signatures in shaping the isotopic composition of atmospheric mercury inputs.

### 3.3 Environmental Factors Controlling Even-MIF Hg Variability in Pyrenean Archives

Mixing models have been recently used to estimate either Hg pollution sources through MDF^46,72,74^ and odd-MIF isotopes,^43^ or deposition pathways through even-MIF isotopes.^22,36,41,47,53^ According to previous studies on Hg deposition in lakes, main inputs come from the atmospheric compartment either by direct deposition or indirectly as a consequence of runoff occurring in the catchment.^8,43,48,53,87–95^

Wet deposition involves the scavenging of gas-phase and aerosol-phase Hg­(II) before their deposition with rainfall and/or snowfall in the lake, whereas gaseous elemental mercury (GEM) dry deposition (Hg(0)) involves surface uptake of Hg(0) directly to the lake by gas exchange or indirectly by runoff or leaching from the watershed subsequent to vegetation uptake. Downcore sediment from Lake Marboré (Figures 2 and 4) displays positive Δ^200^Hg values of 0.09 ± 0.04 ‰ (1σ, *n* = 27), suggesting a significant contribution of wet deposition (precipitation and snow accumulation) over GEM dry deposition consistent with the absence of vegetation in its catchment and the important precipitation rate (2000 mm per year).^59^ In this lake, covered by snow 9–10 months per year, dry deposition is likely limited and may occur through GEM adsorption on snow.^96^ In contrast, even-MIF Δ^200^Hg is lower (0.03 ± 0.05 ‰ [1σ, *n* = 20]) in Lake Estanya and exhibits significant variability, with a decrease in the fraction of Hg coming from GEM dry deposition since the 16th century (higher Δ^200^Hg, *t* test, *p* < 0.05). The relatively constant values in the Estanya record before the 16th century suggest that Hg transport to this site seems to have been dominated by GEM dry deposition through foliar uptake, followed by runoff from the soil catchment. At the onset of the 16th century, both dry and wet depositions increased because of higher atmospheric Hg levels. Nevertheless, the difference in the residence time of Hg in the soil and the atmosphere might explain the observed shift in the Δ^200^Hg signal toward relatively more wet deposition in Lake Estanya. Another possible explanation for this positive shift is the large changes in Lake Estanya catchment occurring since the 16th century due to human activities, with the development of large-scale agricultural activities and the reduction of the forest cover.^67^ These changes in vegetation cover might induce a decrease in the fraction of Hg coming from GEM dry deposition. Finally, changes in local precipitation could also play a role as the 16th–17th centuries included several wetter phases within the Little Ice Age.^64,81^

Despite the uncertainties associated with using modern Δ^200^Hg values from wet deposition as a proxy for reconstructing past mercury sources—and considering analytical limitations—the estimated contribution of wet deposition to total Hg inputs ranges from 16 to 76% in Lake Marboré (median: 57%) and from 0 to 60% in Lake Estanya (median: 33%). This is consistent with the difference in precipitation observed between both lakes, which is higher in Lake Marboré. In addition, Δ^200^Hg in Lake Estanya can be affected not only by climate variability but also by the changes in vegetation surrounding the lake, caused by human activities. Lake Marboré watershed, however, has not experienced large changes in vegetation during the last millennia,^66^ and even-MIF Δ^200^Hg values might be used as a climate proxy. Nevertheless, the exact mechanisms involved are not fully understood. A few years ago, the seasonal variation of Δ^200^Hg in precipitation samples observed by Chen et al.^35^ suggested the possible use of Δ^200^Hg as a tool to monitor related climate effects. More recent studies in the sedimentary record from Lake Titicaca have also successfully used Δ^200^Hg as a climate proxy in South America to differentiate dry and wet periods.^48,97^ Given its remote and stable setting, Lake Marboré provides an ideal platform to explore the potential of Δ^200^Hg as a proxy for regional climate variability. In this line, the influence of climate variability on Lake Marboré isotopic record is supported by the comparison of the reconstructed wet/dry deposition in this lake using Δ^200^Hg with past climate phases identified in the Pyrenees. Samples corresponding to warmer periods (MCA and Roman Warm Period) show distinctively lower Δ^200^Hg values than other samples from the sediment core. The two samples dated in the 12th–13th centuries (1150 and 1270 CE) show lower Δ^200^Hg of 0.04 and −0.01‰, respectively (*t* test, *p* < 0.05), and they correspond to the most arid and warm phase of the last millennium, occurring during the MCA.^81^ During the Roman Warm Period (−250–450 AD), temperatures were also relatively higher in NE Spain^67,98^ and the Marboré samples included in that period–dated −370, 20, 90, and 280 CE–have even-MIF Δ^200^Hg values significantly lower (0.01 to 0.07‰, *t* test, *p* < 0.05). Only one more sample, dated 1930 CE, displays a similar anomaly with lower Δ^200^Hg (0.01‰), and also higher MDF and lower odd-MIF in comparison with closest dated samples. No clear explanation can be provided for this outlier except a local anthropogenic influence, possibly related to the expansion of the Tuquerouye Refuge in 1927 (42°41′N; 0°2′E).^99^ Our isotopic values also show a decreasing trend, although wet deposition is estimated to have remained higher than 50%. Interestingly, the colder and more humid periods as the Dark Ages Cold Period (450–900 AD) and the Little Ice Age (1300–1850 AD) display higher even-MIF Δ^200^Hg values (respectively 0.09 to 0.12 ‰ and 0.07 to 0.13 ‰). The agreement of a consistent even-MIF Δ^200^Hg signal variability recorded in Lake Marboré during the main climatic phases of the last three millennia may suggest Hg isotopes as a promising environmental proxy for paleoclimatic reconstructions. Nevertheless, the isotopic analytical uncertainties and number of measurements impede us to be more conclusive since the mechanism responsible for positive even-MIF (Δ^200^Hg, Δ^204^Hg) in rainfall and snow is not understood yet.^35,41^ Thus, further high-resolution reconstructions are needed to achieve a better comprehension of the relationship between Δ^200^Hg and the climatic variability.

### 3.4 Mercury Isotope Archives in Lakes and Peatlands of the Pyrenees: Disentangling Source Signals from Ecosystem Transformations

Lakes and peatlands are widely recognized as reliable natural archives for reconstructing mercury fluxes, although both systems are influenced by site-specific environmental processes such as emission sources, redox cycling, and postdepositional transformations.^8,10,59^ While ombrotrophic mires may be affected by aerial exposure and diagenetic alteration,^21,95,100–104^ lacustrine sediments generally preserve Hg concentrations more faithfully,^105^ albeit with mixed inputs from both atmospheric deposition and watershed runoff.^53,59,75,95^ Furthermore, metal remobilization and redox changes in the water column may affect Hg concentrations.^95,106^ In addition, internal redox processes in lakes may modulate Hg concentrations^106^

To explore the influence of depositional environment on the Hg isotopic signal, we compared the Lake Marboré record with that from the nearby Estibere peatland^22^ (Figure 5), ∼18 km to the northeast at 2120 m a.s.l. (Figure 1). Both sites are exposed to similar atmospheric Hg sources but differ in precipitation (2000 mm in Marboré vs 1400 mm in Estibere) and ecosystem characteristics. Notably, dry deposition and vegetation uptake dominate Hg accumulation in the peatland, while the alpine lake receives Hg primarily through wet deposition.^22,36^ Our comparison reveals a systematic offset in Δ^199^Hg values, with consistently more positive values in Lake Marboré relative to those in the Estibere mire (Figure 5). Except for the most recent samples (2004 CE for Marboré and 2005 CE for Estibere), the average difference in Δ^199^Hg is 0.33 ± 0.08 ‰ based on interpolated year-by-year values. This offset is robust across the entire record and cannot be explained by temporal variability in atmospheric Hg sources alone. This offset is consistent with the isotopic behavior expected from differing proportions of atmospheric mercury sources, as indicated by the inverse relationship between Hg concentrations and Δ^199^Hg in Lake Marboré. This trend supports a two-endmember mixing scenario, where lower Δ^199^Hg values reflect contributions from anthropogenic emissions, while more negative values are indicative of background atmospheric inputs influenced by wet deposition and photochemical processing. A similar, though less pronounced, pattern is observed in Lake Estanya. These relationships are consistent with observations in other lacustrine and peatland archives (Figure S2), reinforcing the broader applicability of this isotopic framework for distinguishing between Hg sources and depositional processes.

**Figure 5:**
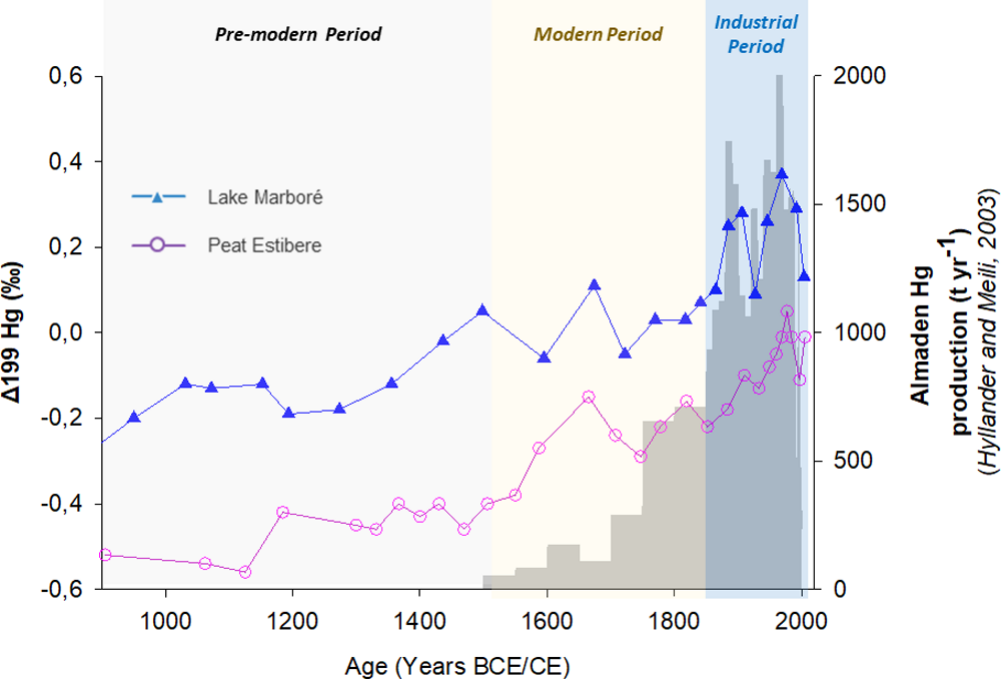
Δ^199^Hg variability during the last millennium in Lake Marboré (blue) and Peat Estibere (from ref 22) (purple) compared with estimations of mercury production in Spanish mines (from Hylander and Meili (2003)). Main historical and climatic phases are also indicated.

Interestingly, while the slopes of Δ^199^Hg–1/[Hg] regressions are broadly similar across lakes and peatlands, their *y*-intercepts diverge systematically, reflecting differences in baseline isotopic signatures. Peatlands tend to cluster around Δ^199^Hg ≈ 0‰, consistent with dominant dry deposition and limited photochemical processing, whereas lakes show more positive intercepts (+0.3 to +0.5‰), suggesting stronger influence from wet deposition and in-lake photoreduction of Hg­(II) (Figure S2A,B). This interpretation is supported by modern measurements showing higher Δ^199^Hg in precipitation (0.71 ± 0.14‰) compared to atmospheric GEM, with values of −0.18 ± 0.07‰ and −0.21 ± 0.03‰ recorded in the Pinet peat record^36^ and Pic du Midi,^51^ respectively (Figure S4A). The higher annual precipitation in Lake Marboré relative to Estibere peatland could thus enhance Hg scavenging via wet deposition and contribute to the observed isotopic offset. Further evidence comes from the Δ^199^Hg vs Δ^201^Hg relationship in Lake Marboré, consistent with surface water photoreduction (Figure S3). The lack of correlation between Δ^199^Hg and Δ^200^Hg supports the idea that odd- and even-MIF are driven by distinct processes (Figure S4).

Despite this isotopic offset, it is important to highlight that both archives show strikingly similar temporal trends, including the post-1500 CE enrichment in Δ^199^Hg that culminates during the Industrial Period (Figure 5). This agreement indicates that both lake and peat records reflect a coherent regional atmospheric Hg signal, reinforcing their value as complementary archives of past mercury deposition. Furthermore, our results indicate that long-range transported Hg, carried in the free troposphere, is more efficiently deposited in high-elevation sites such as Lake Marboré and Estibere mire due to enhanced scavenging by snow and precipitation. This explains why Lake Marboré registers a clearer isotopic imprint of regional emissions (e.g., Almadén), while lowland sites such as Lake Estanya may record a greater influence of local processes.

Altogether, the comparison between lake and peat records in the Pyrenees underlines the dual importance of regional-scale atmospheric forcing and ecosystem-specific processing and illustrates how combined isotopic signals from different archives can enrich our understanding of past mercury deposition and cycling at both local and continental scales. The unique setting of this study—where a remote alpine lake and a peatland are located within the same air mass influence zone—offers an ideal natural laboratory for testing these hypotheses. Importantly, this approach could be extended to other mountainous or remote regions worldwide, where closely situated, yet ecologically distinct, archives may provide complementary perspectives on environmental Hg dynamics over time.

## 4 Conclusions

An integrated retrospective assessment of Hg legacy pollution, evaluating the main pollution sources over time, is needed to improve environmental management policies and pollution risk assessments. In this context, we present the first Late Holocene reconstruction of Hg isotope variability in lake sediments in the Iberian Peninsula using two sedimentary archives (Lake Estanya and Maboré) across an altitudinal gradient in the Southern Central Pyrenees.

Stable Hg isotopes provide valuable insights into long-term biogeochemical cycling, sources, and deposition pathways. The alpine Lake Marboré, with its remote, nonvegetated watershed and minimal human impact, preserves a cleaner isotopic signal of regional atmospheric Hg inputs, while Lake Estanya reflects additional influence from local land-use changes. Despite these differences, both records show consistent long-term trends in mass-dependent fractionation (MDF) and odd mass-independent fractionation (odd-MIF), indicating shared regional sources. The results exhibiting the changes in the mercury stable isotope composition along sediment cores aim to provide new insights into the temporal evolution of major Hg inputs in Pyrenean lacustrine ecosystems. This contributes to refining our understanding of atmospheric Hg sources and historical pollution trends in southwestern Europe.

Isotopic evidence from Lake Marboré indicates that Hg fluxes in high-altitude environments are primarily driven by wet deposition. Its unique environmental features—including an oligotrophic water column, minimal vegetation, and a stable sedimentary archive—allowed us to detect subtle variations in even-MIF (Δ^200^Hg) that align with well-documented Late Holocene climatic phases. This supports the emerging use of Hg isotopes in remote lakes as potential proxies for past climate variability, although further high-resolution studies are needed to consolidate this application.

Importantly, this study also evaluates the persistent isotopic offset between a lacustrine (Lake Marboré) and a nearby ombrotrophic peat archive (Estibere mire), both located in a high alpine environment and subjected to similar atmospheric Hg inputs. The consistent difference in Δ^199^Hg between lake and peat records highlights the role of ecosystem-specific processes—such as deposition pathways, photoreduction, and internal cycling—in shaping isotopic signatures. This dual-record comparison provides a unique natural laboratory to disentangle source signals from depositional effects and contributes to ongoing discussions about the reliability and complementarity of lakes and peatlands as archives of atmospheric mercury.

Overall, the combination of contrasting lake catchments and paired lake–peatland records illustrates how integrating multiple archive types across environmental gradients can enhance our understanding of historical mercury dynamics and improve the reconstruction of both pollution and climate signals at regional to global scales.

## Supplementary Material



## Notes

Additional details on air mass back-trajectory analyses and mercury isotope methods; showing percentages of back trajectories arriving at Lake Estanya and Lake Marboré; reporting Hg isotope compositions for reference materials and replicate lake sediment samples; providing dates, Hg concentrations, Hg accumulation rates, Hg isotope data, and wet deposition percentages for sediment cores; depicting frequency maps of air mass back trajectories; and presenting Hg isotopic relationships in lake sediments and comparisons with other environmental archives (PDF)

## ◆

CEA, DES, IRESNE/DEC/SESC/LARC, Centre de Cadarache, 13108 Saint-Paul-lez-Durance CEDEX, France

## References

[1] Arcagni M. (2018). Species-and habitat-specific bioaccumulation of total mercury and
methylmercury in the food web of a deep oligotrophic lake. Sci. Total Environ..

[2] Anual Z. F. (2018). Mercury and risk assessment
from consumption of crustaceans, cephalopods
and fish from West Peninsular Malaysia. Microchemical
journal.

[3] Xu Q. (2018). Bioaccumulation
characteristics of mercury in fish in the Three Gorges
Reservoir. China. Environmental Pollution.

[4] Sundseth K., Pacyna J. M., Pacyna E. G., Pirrone N., Thorne R. J. (2017). Global
sources and pathways of mercury in the context of human health. International journal of environmental research and public
health.

[5] UNEP Global Mercury Assessment 2013: Sources, Emissions, Releases and Environmental Transport; UNEP Chemicals Branch: Geneva, Switzerland, 2013.

[6] Obrist D. (2018). A review of global environmental
mercury processes in response to
human and natural perturbations: Changes of emissions, climate, and
land use. Ambio.

[7] Brocza F. M., Rafaj P., Sander R., Wagner F., Jones J. M. (2024). Global
scenarios of anthropogenic mercury emissions. Atmos. Chem. Phys..

[8] Driscoll C. T., Mason R. P., Chan H. M., Jacob D. J., Pirrone N. (2013). Mercury as
a global pollutant: sources, pathways, and effects. Environ. Sci. Technol..

[9] Amos H. M. (2015). Observational and modeling constraints on global anthropogenic enrichment
of mercury. Environ. Sci. Technol..

[10] Cooke C. A., Martínez-Cortizas A., Bindler R., Gustin M. S. (2020). Environmental
archives of atmospheric Hg deposition–A review. Sci. Total Environ..

[11] Ariya P. A. (2015). Mercury physicochemical
and biogeochemical transformation in the
atmosphere and at atmospheric interfaces: A review and future directions. Chem. Rev..

[12] Saiz-Lopez A. (2018). Photoreduction of gaseous oxidized mercury changes global atmospheric
mercury speciation, transport and deposition. Nat. Commun..

[13] Saiz-Lopez A. (2019). Gas-phase photolysis
of Hg (I) radical species: A new atmospheric
mercury reduction process. J. Am. Chem. Soc..

[14] Hylander L. D., Meili M. (2003). 500 years of mercury
production: global annual inventory by region
until 2000 and associated emissions. Science
of The Total Environment.

[15] Engstrom D. R. (2014). Atmospheric Hg Emissions from Preindustrial
Gold and Silver Extraction
in the Americas: A Reevaluation from Lake-Sediment Archives. Environ. Sci. Technol..

[16] Corella J. P. (2021). Recent and historical pollution legacy in high altitude Lake Marboré
(Central Pyrenees): A record of mining and smelting since pre-Roman
times in the Iberian Peninsula. Science of The
Total Environment.

[17] Taylor V. F., Landis J. D., Janssen S. E. (2022). Tracing
the sources and depositional
history of mercury to coastal northeastern US lakes. Environmental Science: Processes & Impacts.

[18] Serrano O., Martínez-Cortizas A., Mateo M., Biester H., Bindler R. (2013). Millennial scale impact
on the marine biogeochemical
cycle of mercury from early mining on the Iberian Peninsula. Global Biogeochemical Cycles.

[19] Cossa D. (2023). Mercury deposition in the Eastern Mediterranean: Modern fluxes in
the water column and Holocene accumulation rates in abyssal sediment. Chem. Geol..

[20] Li C. (2020). Unequal anthropogenic enrichment of mercury in Earth’s
northern
and southern hemispheres. ACS Earth and Space
Chemistry.

[21] Martinez-Cortizas A., Pontevedra-Pombal X., García-Rodeja E., Nóvoa-Munoz J. C., Shotyk W. (1999). Mercury in a Spanish Peat Bog: Archive of Climate Change
and Atmospheric Metal Deposition. Science.

[22] Enrico M. (2017). Holocene atmospheric
mercury levels reconstructed from peat bog mercury
stable isotopes. Environ. Sci. Technol..

[23] Beal S. A., Osterberg E. C., Zdanowicz C. M., Fisher D. A. (2015). Ice Core Perspective
on Mercury Pollution during the Past 600 Years. Environ. Sci. Technol..

[24] Schuster P. F. (2002). Atmospheric Mercury Deposition during the Last 270 Years: A Glacial
Ice Core Record of Natural and Anthropogenic Sources. Environ. Sci. Technol..

[25] Eyrikh S. (2017). A 320 Year Ice-Core Record of Atmospheric Hg Pollution in the Altai,
Central Asia. Environ. Sci. Technol..

[26] Segato D. (2023). Arctic mercury flux
increased through the Last Glacial Termination
with a warming climate. Nat. Geosci..

[27] Jitaru P. (2009). Atmospheric depletion
of mercury over Antarctica during glacial periods. Nature Geoscience.

[28] Pérez-Rodríguez M. (2018). The role of climate: 71 ka of atmospheric mercury deposition in the
Southern Hemisphere recorded by Rano Aroi Mire, Easter Island (Chile). Geosciences.

[29] Blum J. D., Sherman L. S., Johnson M. W. (2014). Mercury
isotopes in earth and environmental
sciences. Annual Review of Earth and Planetary
Sciences.

[30] Kwon S. Y. (2020). Mercury stable isotopes for monitoring the effectiveness of the Minamata
Convention on Mercury. Earth-Science Reviews.

[31] Tsui M. T.-K., Blum J. D., Kwon S. Y. (2020). Review
of stable mercury isotopes
in ecology and biogeochemistry. Science of The
Total Environment.

[32] Sherman L. S., Blum J. D., Keeler G. J., Demers J. D., Dvonch J. T. (2012). Investigation
of Local Mercury Deposition from a Coal-Fired Power Plant Using Mercury
Isotopes. Environ. Sci. Technol..

[33] Perrot V. (2016). Natural Hg isotopic
composition of different Hg compounds in mammal
tissues as a proxy for in vivo breakdown of toxic methylmercury. Metallomics.

[34] Bergquist B. A., Blum J. D. (2007). Mass-dependent and-independent fractionation of Hg
isotopes by photoreduction in aquatic systems. Science.

[35] Chen J., Hintelmann H., Feng X., Dimock B. (2012). Unusual fractionation
of both odd and even mercury isotopes in precipitation from Peterborough,
ON, Canada. Geochim. Cosmochim. Acta.

[36] Enrico M. (2016). Atmospheric mercury
transfer to peat bogs dominated by gaseous elemental
mercury dry deposition. Environ. Sci. Technol..

[37] Cai H., Chen J. (2016). Mass-independent fractionation
of even mercury isotopes. Science bulletin.

[38] Blum J. D., Johnson M. W. (2017). Recent developments
in mercury stable isotope analysis. Reviews
in Mineralogy and Geochemistry.

[39] Saiz-Lopez A. (2025). Role of the stratosphere in the global mercury cycle. Sci. Adv..

[40] Lee J. H. (2021). Spatiotemporal
Characterization of Mercury Isotope Baselines and
Anthropogenic Influences in Lake Sediment Cores. Global Biogeochemical Cycles.

[41] Kurz A. Y., Blum J. D., Washburn S. J., Baskaran M. (2019). Changes in the mercury
isotopic composition of sediments from a remote alpine lake in Wyoming,
USA. Science of The Total Environment.

[42] Cooke C. A. (2013). Use and legacy of mercury
in the Andes. Environ.
Sci. Technol..

[43] Guédron S. (2016). A hundred
year record of industrial and urban development in French
Alps combining Hg accumulation rates and isotope composition in sediment
archives from Lake Luitel. Chem. Geol..

[44] Jackson T. A. (2019). Stratigraphic
variations in the δ 201 Hg/δ 199 Hg ratio of mercury in
sediment cores as historical records of methylmercury production in
lakes. Journal of Paleolimnology.

[45] Jackson T. A., Muir D. C., Vincent W. F. (2004). Historical
variations in the stable
isotope composition of mercury in Arctic lake sediments. Environ. Sci. Technol..

[46] Yin R., Lepak R. F., Krabbenhoft D. P., Hurley J. P. (2016). Sedimentary records
of mercury stable isotopes in Lake MichiganMercury isotopic records
in sediment cores of Lake Michigan. Elementa
Sci. Anthropocene.

[47] Yin R. (2016). Historical records of
mercury stable isotopes in sediments of Tibetan
lakes. Sci. Rep..

[48] Jiskra M. (2022). Climatic Controls on
a Holocene Mercury Stable Isotope Sediment Record
of Lake Titicaca. ACS Earth and Space Chemistry.

[49] Sun R. (2022). Mercury Isotope Variations
in Lake Sediment Cores in Response to
Direct Mercury Emissions from Non-Ferrous Metal Smelters and Legacy
Mercury Remobilization. Environ. Sci. Technol..

[50] Hughes-Allen L. (2024). A 14,000-Year Sediment
Record of Mercury Accumulation and Isotopic
Signatures From Lake Malaya Chabyda (Siberia). *Journal of
Geophysical Research*. Biogeosciences.

[51] Fu X., Marusczak N., Wang X., Gheusi F., Sonke J. E. (2016). Isotopic
composition of gaseous elemental mercury in the free troposphere of
the Pic du Midi Observatory, France. Environ.
Sci. Technol..

[52] Obrist D. (2017). Tundra uptake of atmospheric elemental mercury drives Arctic mercury
pollution. Nature.

[53] Chen J. (2016). Isotopic evidence for
distinct sources of mercury in lake waters
and sediments. Chem. Geol..

[54] Demers J. D., Blum J. D., Zak D. R. (2013). Mercury
isotopes in a forested ecosystem:
Implications for air-surface exchange dynamics and the global mercury
cycle. Global Biogeochemical Cycles.

[55] Zdanowicz C. M. (2016). Historical variations
of mercury stable isotope ratios in Arctic
glacier firn and ice cores. Global Biogeochemical
Cycles.

[56] Leblanc M., Morales J., Borrego J., Elbaz-Poulichet F. (2000). 4,500-year-old
mining pollution in southwestern Spain: long-term implications for
modern mining pollution. Econ. Geol..

[57] Morellón M. (2009). Lateglacial and Holocene
palaeohydrology in the western Mediterranean
region: The Lake Estanya record (NE Spain). Quaternary Science Reviews.

[58] Valero-Garcés, B. L. Dinámica glacial, clima y vegetación en el Parque Nacional de Ordesa y Monte Perdido durante el Holoceno. In Proyectos de Investigación en Parques Nacionales; MAGRAMA, 2013.

[59] Corella J. (2018). Trace metal enrichment during the Industrial Period recorded across
an altitudinal transect in the Southern Central Pyrenees. Science of The Total Environment.

[60] Morellón M. (2009). Late Quaternary deposition
and facies model for karstic Lake Estanya
(NE Spain). Sedimentology.

[61] Oliva-Urcia B. (2018). Last deglaciation and
Holocene environmental change at high altitude
in the Pyrenees: the geochemical and paleomagnetic record from Marboré
Lake (N Spain). Journal of paleolimnology.

[62] Nicolás-Martínez P. M. (1981). Morfología
del circo de Tucarroya (Macizo de Monte Perdido, Pirineo aragonés). Cuadernos de Investigación Geográfica.

[63] Sánchez-España J. (2018). Limnochemistry of the remote, high mountain Lake Marboré (Ordesa
and Monte Perdido National Park, Central Pyrenees): Stratification
dynamics and trace metal anomalies. Limnetica.

[64] Morellón M. (2011). Climate changes and human activities recorded
in the sediments of
Lake Estanya (NE Spain) during the Medieval Warm Period and Little
Ice Age. Journal of Paleolimnology.

[65] Leunda M., Gil-Romera G., Daniau A.-L., Benito B. M., González-Sampériz P. (2020). Holocene fire
and vegetation dynamics in the Central Pyrenees (Spain). Catena.

[66] Leunda M. (2017). The Late-Glacial and Holocene Marboré Lake sequence
(2612
m asl, Central Pyrenees, Spain): testing high altitude sites sensitivity
to millennial scale vegetation and climate variability. Global and planetary change.

[67] González-Sampériz P. (2017). Environmental and climate change in the southern Central Pyrenees
since the Last Glacial Maximum: A view from the lake records. Catena.

[68] Draxler, R. R. ; Stunder, B. ; Rolph, G. ; Taylor, J. NOAA Air Resources Laboratory, Silver Spring, MD. December 1997, revised January 2009, 2009. http://www.arl.noaa.gov/documents/reports/hysplit_user_guide.pdf.

[69] Stein A. (2015). NOAA’s HYSPLIT
atmospheric transport and dispersion modeling
system. Bulletin of the American Meteorological
Society.

[70] Dee D. P. (2011). The
ERA-Interim reanalysis: Configuration and performance of the
data assimilation system. Quarterly Journal
of the royal meteorological society.

[71] Givelet N., Roos-Barraclough F., Shotyk W. (2003). Predominant anthropogenic sources
and rates of atmospheric mercury accumulation in southern Ontario
recorded by peat cores from three bogs: comparison with natural “background”
values (past 8000 years). Journal of Environmental
Monitoring.

[72] Jiménez-Moreno M. (2016). Sources and fate of mercury pollution in Almadén mining district
(Spain): Evidences from mercury isotopic compositions in sediments
and lichens. Chemosphere.

[73] Blum J. D., Bergquist B. A. (2007). Reporting
of variations in the natural isotopic composition
of mercury. Anal. Bioanal. Chem..

[74] Barre J. P. (2018). Multi-element isotopic
signature (C, N, Pb, Hg) in epiphytic lichens
to discriminate atmospheric contamination as a function of land-use
characteristics (Pyrénées-Atlantiques, SW France). Environ. Pollut..

[75] Corella J. P. (2017). 700 years reconstruction of mercury and lead
atmospheric deposition
in the Pyrenees (NE Spain). Atmospheric environment.

[76] Higueras P., Mansilla L., Lorenzo S., Esbrí J. (2011). The Almadén
mercury mining district. Hist. Res. Miner. Resour..

[77] Zhou J., Obrist D. (2021). Global Mercury Assimilation
by Vegetation. Environ. Sci. Technol..

[78] Chételat J. (2015). Mercury in freshwater
ecosystems of the Canadian Arctic: recent advances
on its cycling and fate. Sci. Total Environ..

[79] Pérez-Rodríguez M., Biester H., Aboal J. R., Toro M., Martínez
Cortizas A. (2019). Thawing of snow and ice caused extraordinary high and
fast mercury fluxes to lake sediments in Antarctica. Geochim. Cosmochim. Acta.

[80] Marusczak N. (2011). Total mercury and methylmercury
in high altitude surface snow from
the French Alps. Science of the total environment.

[81] Morellón M. (2012). A multi-proxy perspective
on millennium-long climate variability
in the Southern Pyrenees. Climate of the Past.

[82] Corella J. P. (2012). The 1.5-ka varved record
of Lake Montcortès (southern Pyrenees,
NE Spain). Quat. Res..

[83] Lepak R. F. (2020). Resolving atmospheric
mercury loading and source trends from isotopic
records of remote North American lake sediments. Environ. Sci. Technol..

[84] Gómez M. G. (2007). Exposure to mercury in the mine of Almaden. Occup. Environ. Med..

[85] Gray J. E., Pribil M. J., Higueras P. L. (2013). Mercury
isotope fractionation during
ore retorting in the Almadén mining district. Spain. Chemical Geology.

[86] Sun R. (2013). Mercury stable isotope fractionation in six utility
boilers of two
large coal-fired power plants. Chem. Geol..

[87] Cohen M. D. (2016). Modeling the global
atmospheric transport and deposition of mercury
to the Great Lakes. Elementa Sci. Anthropocene.

[88] Guédron S. (2017). Mercury contamination
level and speciation inventory in Lakes Titicaca
& Uru-Uru (Bolivia): Current status and future trends. Environmental pollution.

[89] Hammerschmidt C. R., Fitzgerald W. F. (2006). Methylmercury in freshwater fish
linked to atmospheric
mercury deposition. Environ. Sci. Technol..

[90] Mason R. P., Sullivan K. A. (1997). Mercury in lake
Michigan. Environ.
Sci. Technol..

[91] Meuleman, C. ; Leermakers, M. ; Baeyens, W. In Mercury as a Global Pollutant, Proceedings of the Third International Conference held in Whistler, British Columbia, July 10–14, 1994; Springer, 1994; pp 539–551.

[92] Økelsrud A., Lydersen E., Fjeld E. (2016). Biomagnification of mercury and selenium
in two lakes in southern Norway. Sci. Total
Environ..

[93] Rolfhus K. (2003). Distribution and fluxes of total and methylmercury in Lake Superior. Environ. Sci. Technol..

[94] Wiener J. (2006). Mercury in soils, lakes, and fish in Voyageurs National Park (Minnesota):
importance of atmospheric deposition and ecosystem factors. Environ. Sci. Technol..

[95] Biester H., Bindler R., Martinez-Cortizas A., Engstrom D. R. (2007). Modeling the past
atmospheric deposition of mercury using natural archives. Environ. Sci. Technol..

[96] Douglas T. A., Blum J. D. (2019). Mercury isotopes reveal atmospheric gaseous mercury
deposition directly to the Arctic coastal snowpack. Environmental Science & Technology Letters.

[97] Xue W. (2023). Climatic regulation
of atmospheric mercury deposition: Evidence from
mercury isotopes in an alpine peat core. Geology.

[98] Corella J. P. (2013). A 2500-year multi-proxy
reconstruction of climate change and human
activities in northern Spain: The Lake Arreo record. Palaeogeography, Palaeoclimatology, Palaeoecology.

[99] Stampa E. M. D. P. (2014). Ordesa:
del valle perdido al símbolo patrimonial. Ería.

[100] Martínez Cortizas A., Peiteado Varela E., Bindler R., Biester H., Cheburkin A. (2012). Reconstructing
historical Pb and Hg pollution in NW Spain using multiple cores from
the Chao de Lamoso bog (Xistral Mountains). Geochim. Cosmochim. Acta.

[101] Biester H. (2002). Elevated mercury accumulation in a peat
bog of the
Magellanic Moorlands, Chile (53°S)–an anthropogenic signal
from the Southern Hemisphere. Earth and Planetary
Science Letters.

[102] Biester H., Martinez-Cortizas A., Birkenstock S., Kilian R. (2003). Effect of peat decomposition and mass loss on historic
mercury records in peat bogs from Patagonia. Environ. Sci. Technol..

[103] Bandara S., Froese D. G., St. Louis V. L., Cooke C. A., Calmels F. (2019). Postdepositional
mercury mobility
in a permafrost peatland from central Yukon, Canada. ACS Earth Space Chem..

[104] Osterwalder S. (2017). Mercury evasion from
a boreal peatland shortens
the timeline for recovery from legacy pollution. Sci. Rep..

[105] Rydberg J. (2008). Assessing the stability
of mercury and methylmercury
in a varved lake sediment deposit. Environ.
Sci. Technol..

[106] Duval B. (2023). Dynamics, distribution,
and transformations of mercury
species from pyrenean high-altitude lakes. Environmental
Research.

