# Supplementary material for: Mercury Isotope Variability in Pyrenean Lake Sediments during the Late Holocene: Sources, Deposition, and Environmental Controls
Source: ACS Earth Space Chem. 2025 May 13;9(6):1488–500. doi: 10.1021/acsearthspacechem.4c00402 (PMC12186775; doi:10.1021/acsearthspacechem.4c00402)
Supplement: Supplementary file 1 [file sp4c00402_si_001.pdf]

Supporting Information

Mercury Isotope Variability in Pyrenean Lake Sediments During the Late Holocene: Sources, Deposition, and Environmental Controls

Bastien Duval[a,b, c], Juan Pablo Corella[*d], Maxime Enrico[a], Alfonso Saiz-Lopez[e], Carlos A. Cuevas[e], Jose A. Adame[f], Rocío Millán[g], Maria J. Sierra[g], Sylvain Bérail[a], Blas L. Valero-Garcés[h], Alberto de Diego[b], Mario Morellón[i], Javier Rodríguez-Alonso[g], David Amouroux[a]

[a]*Universite de Pau et des Pays de l'Adour / E2S UPPA, CNRS, Institut des Sciences Analytiques et de Physico-chimie pour l'Environnement et les Materiaux, UMR5254, Helioparc, 64053 Pau, France*

[b]*Kimika Analitikoa Saila, Universidad del País Vasco/Euskal Herriko Unibertsitatea UPV/EHU, Sarriena Auzoa z/g, 48940 Leioa, Spain*

[c]Present-day affiliation*: CEA, DES, IRESNE/DEC/SESC/LARC, Centre de Cadarache, 13108 CEDEX, Saint-Paul-lez-Durance, France*

[d]*National Museum of Natural Sciences-CSIC, Serrano 115 bis, 28006, Madrid, Spain.*

[e]*Department of Atmospheric Chemistry and Climate, Institute of Physical Chemistry Blas Cabrera, CSIC, Serrano 119, 28006 Madrid, Spain*

[f]*Atmospheric Sounding Station. El Arenosillo. National Institute for Aerospace Technology (INTA). Mazagón7Huelva. Spain.*

[g]*CIEMAT, Department of the Environment (DMA), Avenida Complutense 40, E-28040 Madrid, Spain*

[h]*Pyrenean Institute of Ecology, CSIC, Avda Montañana 1005, 50059 Zaragoza, Spain*

[i]*Departament of Geodynamics, Stratigraphy and Paleontology, Faculty of Geological Sciences, Complutense University of Madrid, Calle José Antonio Nováis 12, 28040 Madrid, Spain*

**Corresponding authors*

Juan Pablo Corella E-mail: pablo.corella@mncn.csic.es

Recent air masses back- trajectories evolution in the Central Pyrenees

The computed air masses during the last decades showed that most of the air masses arriving at the two investigated lakes originated from South-western Europe. Previous back-trajectory analyses in Ordesa National Park, where Lake Marboré is located, showed that 50 % of air masses come from SW France and the Ebro valley, while Central and Western Spain, Southern Spain and Northern Africa contributed to 16.4%, 17.4%, and 3%, respectively [1]. These results were assumed to be representative of atmospheric circulation dynamics during the Late Holocene [1]. Our back-trajectories analyses performed in Lake Estanya show that the predominant source of air masses reaching Lake Estanya is restricted to the Ebro basin, accounting for 99.7 % of all the trajectories that reach the lake have previously passed through the Ebro valley and, secondarily from South-western France (41.5 %) (Supporting Information: Table S1 contains percentages of back-trajectories analyses). Our modelling results also showed that air masses travelling long distances usually move in the upper layers, i.e. emissions from the surface in the atmospheric boundary layer reach the free troposphere and then are transported by the westerly flows. Thus, high altitude Lake Marboré would therefore record the impact of long-range transport of trace metals-bearing air masses travelling in the free troposphere from South-western Europe, while Lake Estanya would record more local processes occurring mostly in the Ebro Basin. Nevertheless, still 21.7 % of the total back-trajectories arriving at Lake Estanya passed over the Almadén region (Fig. S1, Table S1) clearly indicating that Hg emissions related to major mining phases would also reach low elevation Lake Estanya. The isotopic signature of pollution sources would be more efficiently recorded in Lake Marbore because of its high elevation and higher precipitation regime that would ease atmospheric Hg scavenging.

Hg isotopic composition analyses

Hg isotopic compositions of samples are reported using delta notation according to the following equation (eq 1):

$$(1) \qquad \delta^{xxx}Hg\ (‰) = \left[\left(\frac{\left(\frac{{}^{xxx}_{0}Hg}{{}^{198}_{0}Hg}\right)_{sample}}{\left(\frac{{}^{xxx}_{0}Hg}{{}^{198}_{0}Hg}\right)_{NIST\ 3133}}\right) - 1\right] \times 1000$$

where xxx is the mass of each Hg isotope between 199 and 204, ^{198}Hg is used as a reference because it is one of the lighters Hg isotopes (^{196}Hg has a too-small abundance).

Mass Independent Fractionation (MIF) anomalies are expressed with the Δ notation, quantifying the difference between the measured isotope ratio $\delta^{xxx}Hg$ and the theoretical value $\delta^{xxx}Hg$, calculated based on the Mass Dependent Fractionation (MDF) fractionation laws. As for the δ notation, we report Hg MIF anomalies as proposed by Blum and Bergquist [2] to ensure data comparison (eq 2, 3, 4,5):

$$(2) \quad \Delta^{199}Hg = \delta^{199}Hg_{exp} - \delta^{202}Hg_{theo} \times 0.2520$$

$$(3) \quad \Delta^{200}Hg = \delta^{200}Hg_{exp} - \delta^{202}Hg_{theo} \times 0.5024$$

$$(4) \quad \Delta^{201}Hg = \delta^{201}Hg_{exp} - \delta^{202}Hg_{theo} \times 0.7520$$

$$(5) \quad \Delta^{204}Hg = \delta^{204}Hg_{exp} - \delta^{202}Hg_{theo} \times 1.4930$$

Table S1: Back trajectories percentages passing over different regions and arriving Lake Estanya and Lake Marboré area.

**Region**	**Longitude**	**Latitude**	**Percentage of back-trajectories**	
			**Lake Estanya**	**Lake Marboré**
SW France	42.83N - 45.83N	1.83W - 0.83E	41.48	51.9
Ebro valley	40.00N - 42.50E	1.83W - 2.83E	99.77	52.1
Central and western Spain	40.00N - 42.00N	10.00W - 2.5W	21.67	16.4
Southern Spain	36.00N - 40.00N	10.00W - 1.00E	24.61	17.4
Northern Africa	30.00N - 36.00N	10.00W - 5.00E	4.11	3.0

Table S2. Mean values (± 2SD) of Hg isotopic composition obtained for reference materials NIST-8610 (UM-Almadén), IAEA-405 (estuarine sediment) and NIST-1944 (marine sediment), and for triplicate Hg extraction for lakes Marboré and Estanya.

Sample	*Reference*	*n*	$\delta^{204}Hg$ ‰	$\delta^{202}Hg$ ‰	$\delta^{201}Hg$ ‰	$\delta^{200}Hg$ ‰	$\delta^{199}Hg$ ‰	$\Delta^{204}Hg$ ‰	$\Delta^{201}Hg$ ‰	$\Delta^{200}Hg$ ‰	$\Delta^{199}Hg$ ‰
**NIST RM 8610**	This study	32	-0.78±0.18	-0.52±0.12	-0.42±0.12	-0.26±0.10	-0.12±0.14	0.00±0.14	-0.03±0.07	0.00±0.06	-0.01±0.12
	Reference values		-0.82±0.07	-0.56±0.03	-0.46±0.02	-0.27±0.01	-0.17±0.01	-	-0.04±0.01	0.00±0.01	-0.03±0.02
**IAEA 405**	This study	7	-0.57±0.15	-0.39±0.09	-0.30±0.13	-0.20±0.08	-0.11±0.06	0.02±0.08	0.00±0.08	0.00±0.05	-0.01±0.06
	[3]	14	-0.62±0.21	-0.41±0.16	-0.31±0.19	-0.19±0.12	-0.12±0.11	-	-0.01±0.09	0.01±0.06	-0.02±0.08
	[4]	14	-0.50±0.17	-0.30±0.11	-0.29±0.07	-0.22±0.11	-0.17±0.15	-	-0.06±0.06	-0.07±0.13	-0.10±0.15
**NIST 1944**	IPREM (2011-2015)	15	-0.68±0.17	-0.44±0.14	-0.32±0.18	-0.23±0.13	-0.11±0.12	-	0.01±0.12	-0.01±0.10	-0.00±0.11
	[5]	9	-	-0.44±0.12	-0.34±0.08	-0.22±0.05	-0.10±0.04	-	-0.01±0.05	0.00±0.03	0.01±0.04
	[6]	3	-	-0.48±0.29	-0.38±0.18	-0.21±0.23	-0.10±0.02	-	-0.01±0.04	0.04±0.09	0.02±0.05
	[7]	5	-	-0.45±0.06	-	-	-	-	-	-	-0.03±0.02
	[8]	10	-	-0.42±0.07	-	-	-	-	-0.02±0.01	-	-0.02±0.01
**Maboré**	This study	3	-1.07±0.11	-0.59±0.15	-0.48±0.10	-0.21±0.10	-0.20±0.10	-0.18±0.18	-0.03±0.05	0.09±0.07	-0.05±0.09
**Estanya**	This study	3	-2.91±0.15	-1.98±0.17	-1.57±0.16	-1.00±0.08	-0.57±0.05	0.04±0.14	-0.08±0.03	-0.01±0.01	-0.07±0.08

Table S3. Dates, total Hg concentrations, HgARs, Hg isotopic composition and percentage of wet deposition (calculated using Δ^{200}HgWet and Δ^{200}Hg Dry end-members) for the sediment cores of Lake Marboré and Lake Estanya.

Lake	*Sample*	*Date* *year*	*Hg* *ng g⁻¹*	*HgARs* *µg m⁻² y⁻¹*	*δ^{204}Hg* *‰*	*δ^{202}Hg* *‰*	*δ^{201}Hg* *‰*	*δ^{200}Hg* *‰*	*δ^{199}Hg* *‰*	*Δ^{204}Hg* *‰*	*Δ^{201}Hg* *‰*	*Δ^{200}Hg* *‰*	*Δ^{199}Hg* *‰*	*Wet deposition calculated* *%*
Lake Marboré	MAR11-1A-1U-1, 8-9 cm	2004	58	40	-0.96	-0.51	-0.15	-0.18	0.00	-0.19	0.24	0.08	0.13	50
Lake Marboré	MAR11-1A-1G-1, 10-11 cm	1992	64	49	-1.15	-0.63	-0.24	-0.22	0.13	-0.21	0.23	0.10	0.29	58
Lake Marboré	MAR11-1A-1G-1, 12-13 cm	1969	106	70	-1.00	-0.51	0.00	-0.14	0.24	-0.24	0.38	0.12	0.37	65
Lake Marboré	MAR11-1A-1G-1, 14-15 cm	1946	53	39	-1.14	-0.64	-0.25	-0.21	0.09	-0.19	0.23	0.11	0.26	61
Lake Marboré	MAR11-1A-1U-1, 14-15 cm	1927	53	39	-0.04	0.11	0.22	0.07	0.12	-0.20	0.13	0.01	0.09	23
Lake Marboré	MAR11-1A-1U-1, 16-17 cm	1906	67	54	-0.74	-0.37	-0.06	-0.04	0.19	-0.18	0.22	0.15	0.28	76
Lake Marboré	MAR11-1A-1U-1, 18-19 cm	1885	58	44	-0.55	-0.23	0.00	0.01	0.19	-0.20	0.17	0.13	0.25	67
Lake Marboré	MAR11-1A-1U-1, 20-21 cm	1865	67	48	-0.52	-0.27	-0.13	-0.01	0.04	-0.12	0.07	0.13	0.10	68
Lake Marboré	MAR11-1A-1U-1, 22-23 cm	1841	50	30	-0.62	-0.35	-0.17	-0.08	-0.01	-0.10	0.09	0.10	0.07	56
Lake Marboré	MAR11-1A-1U-1, 24-25 cm	1817	37	25	-1.24	-0.74	-0.54	-0.27	-0.15	-0.13	0.01	0.10	0.03	57
Lake Marboré	MAR11-1A-1U-1, 28-29 cm	1770	38	22	-0.75	-0.38	-0.34	-0.08	-0.06	-0.19	-0.05	0.11	0.03	63
Lake Marboré	MAR11-1A-1U-1, 32-33 cm	1722	32	23	-1.07	-0.59	-0.48	-0.21	-0.20	-0.18	-0.03	0.09	-0.05	54
Lake Marboré	MAR11-1A-1U-1, 36-37 cm	1674	26	15	-0.90	-0.52	-0.27	-0.13	-0.02	-0.13	0.11	0.13	0.11	70
Lake Marboré	MAR11-1A-1U-1, 40-41 cm	1595	26	17	-0.88	-0.39	-0.31	-0.13	-0.16	-0.30	-0.02	0.07	-0.06	45
Lake Marboré	MAR11-1A-1U-1, 42-43 cm	1498	25	18	-0.68	-0.35	-0.35	-0.08	-0.04	-0.15	-0.09	0.10	0.05	56
Lake Marboré	MAR11-1A-1U-2, 15-16 cm	1436	22	14	-1.66	-0.99	-0.84	-0.39	-0.27	-0.18	-0.10	0.11	-0.02	60
Lake Marboré	MAR11-1A-1U-2, 19-20 cm	1355	20	14	-1.61	-1.03	-0.87	-0.40	-0.38	-0.08	-0.10	0.11	-0.12	63
Lake Marboré	MAR11-1A-1U-2, 23-24 cm	1273	25	13	-2.12	-1.23	-1.19	-0.63	-0.49	-0.28	-0.27	-0.01	-0.18	16
Lake Marboré	MAR11-1A-1U-2, 29-30 cm	1152	27	16	-1.99	-1.33	-1.13	-0.63	-0.46	0.00	-0.13	0.04	-0.12	33
Lake Marboré	MAR11-1A-1U-2, 45-46 cm	829	22	14	-2.18	-1.31	-1.06	-0.54	-0.43	-0.22	-0.07	0.12	-0.09	66
Lake Marboré	MAR11-1A-1U-2, 57-58 cm	600	19	14	-1.71	-1.04	-0.90	-0.43	-0.35	-0.16	-0.12	0.09	-0.09	53
Lake Marboré	MAR11-1A-1U-2, 67-68 cm	438	23	14	-2.24	-1.46	-1.13	-0.61	-0.44	-0.05	-0.03	0.12	-0.07	67

Lake Marboré	MAR11-1A-1U-2, 77-78 cm	278	20	17	-2.64	-1.73	-1.36	-0.80	-0.47	-0.05	-0.05	0.07	-0.04	45
Lake Marboré	MAR11-1A-1U-2, 89-90 cm	85	19	14	-3.12	-1.99	-1.67	-0.96	-0.66	-0.14	-0.17	0.04	-0.16	36
Lake Marboré	MAR11-1A-1U-2, 93-94 cm	20	31	18	-2.94	-1.83	-1.51	-0.91	-0.58	-0.20	-0.13	0.01	-0.12	24
Lake Marboré	MAR11-1A-1U-2, 117-118 cm	-366	24	13	-2.17	-1.35	-1.19	-0.62	-0.41	-0.16	-0.17	0.06	-0.07	41
Lake Marboré	MAR11-1A-1U-2, 147-148 cm	-835	19	12	-1.89	-1.17	-1.02	-0.45	-0.34	-0.15	-0.15	0.13	-0.04	70
Lake Estanya	LEG04-1A-1M, 1-6 cm	2001	37	29	-0.93	-0.70	-0.56	-0.30	-0.19	0.12	-0.03	0.05	-0.01	39
Lake Estanya	LEG04-1A-1M, 9-15 cm	1976	29	23	-0.99	-0.62	-0.42	-0.27	-0.17	-0.06	0.05	0.04	-0.01	36
Lake Estanya	LEG04-1A-1K-1, 6-7 cm	1952	79	37	-0.81	-0.45	-0.08	-0.12	0.20	-0.14	0.26	0.11	0.31	60
Lake Estanya	LEG04-1A-1K-1, 7-8 cm	1940	77	54	-0.69	-0.56	-0.25	-0.18	0.10	0.15	0.17	0.10	0.24	59
Lake Estanya	LEG04-1A-1K-1, 12-13 cm	1873	97	51	-0.35	-0.28	-0.11	-0.11	0.14	0.07	0.10	0.03	0.21	31
Lake Estanya	LEG04-1A-1K-1, 16-17 cm	1808	75	48	-1.07	-0.69	-0.37	-0.27	-0.10	1.54	0.15	0.07	0.08	48
Lake Estanya	LEG04-1A-1K-1, 18-19 cm	1776	86	40	-0.61	-0.45	-0.13	-0.17	0.09	0.06	0.21	0.06	0.20	42
Lake Estanya	LEG04-1A-1K-1, 24-25 cm	1687	31	21	-0.98	-0.69	-0.50	-0.26	-0.15	0.06	0.02	0.09	0.02	54
Lake Estanya	LEG04-1A-1K1, 30-31 cm	1612	26	24	-0,72	-0,61	-0,44	-0,32	-0,06	0,19	0,02	-0,01	0,09	14
Lake Estanya	LEG04-1A-1K-1, 41-42 cm	1501	31	24	-1.38	-0.84	-0.61	-0.43	-0.26	-0.12	0.02	-0.01	-0.05	15
Lake Estanya	LEG04-1A-1K-2, 4-5 cm	1269	20	5	-1.20	-0.79	-0.65	-0.42	-0.20	-0.02	-0.06	-0.02	0.00	11
Lake Estanya	LEG04-1A-1K2, 34-35 cm	950	17	6	-0,77	-0,53	-0,56	-0,29	-0,18	0,02	-0,16	-0,03	-0,05	9
Lake Estanya	LEG04-1A-1K-2, 38-39 cm	852	16	4	-1.29	-0.87	-0.64	-0.49	-0.23	0.00	0.01	-0.05	-0.01	0
Lake Estanya	LEG04-1A-1K2, 52-53 cm	324	18	6	-1,08	-0,79	-0,74	-0,34	-0,45	0,09	-0,15	0,06	-0,25	42
Lake Estanya	LEG04-1A-1K2, 58-59 cm	14	17	5	-1,23	-0,83	-0,62	-0,36	-0,22	0,01	0,01	0,06	-0,01	42
Lake Estanya	LEG04-1A-1K2, 68-69 cm	-574	12	3	-1,81	-1,24	-0,80	-0,56	-0,13	0,04	0,13	0,07	0,18	44
Lake Estanya	LEG04-1A-1K-2, 72-73 cm	-825	17	4	-1.22	-0.85	-0.52	-0.47	-0.16	0.04	0.12	-0.04	0.05	2
Lake Estanya	LEG04-1A-1K2, 76-77 cm	-1081	11	3	-1,21	-0,89	-0,56	-0,43	-0,01	0,12	0,11	0,01	0,22	25
Lake Estanya	LEG04-1A-1K-2, 82-83 cm	-1467	24	6	-0.95	-0.72	-0.59	-0.34	-0.16	0.12	-0.05	0.02	0.02	26
Lake Estanya	LEG04-1A-1K-2, 92-93 cm	-2086	15	4	-0.91	-0.71	-0.49	-0.36	-0.02	0.15	0.05	0.00	0.16	19

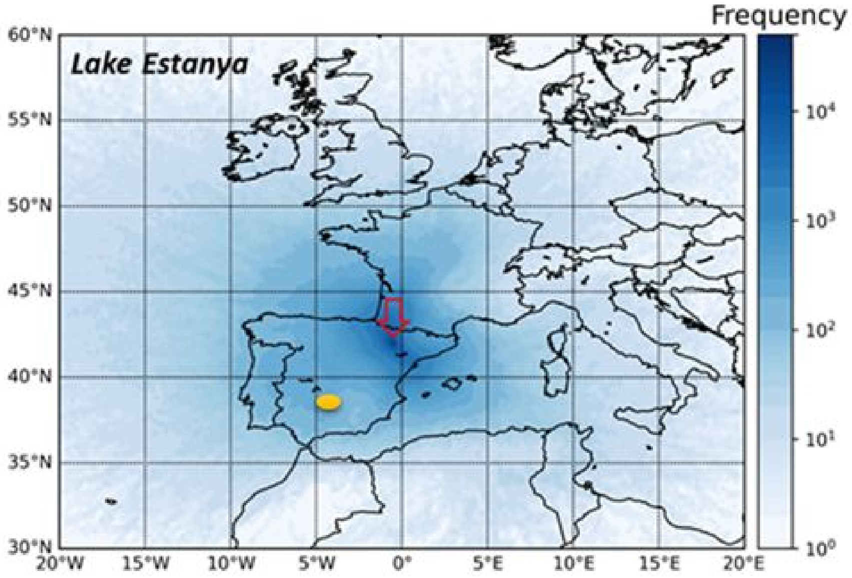

Figure S1: Frequency map of air mass back trajectories for the period 1960-2016 yrs CE arriving to Lake Estanya. Yellow circle: Location of Almadén mines, red arrow: location of Lake Estanya.

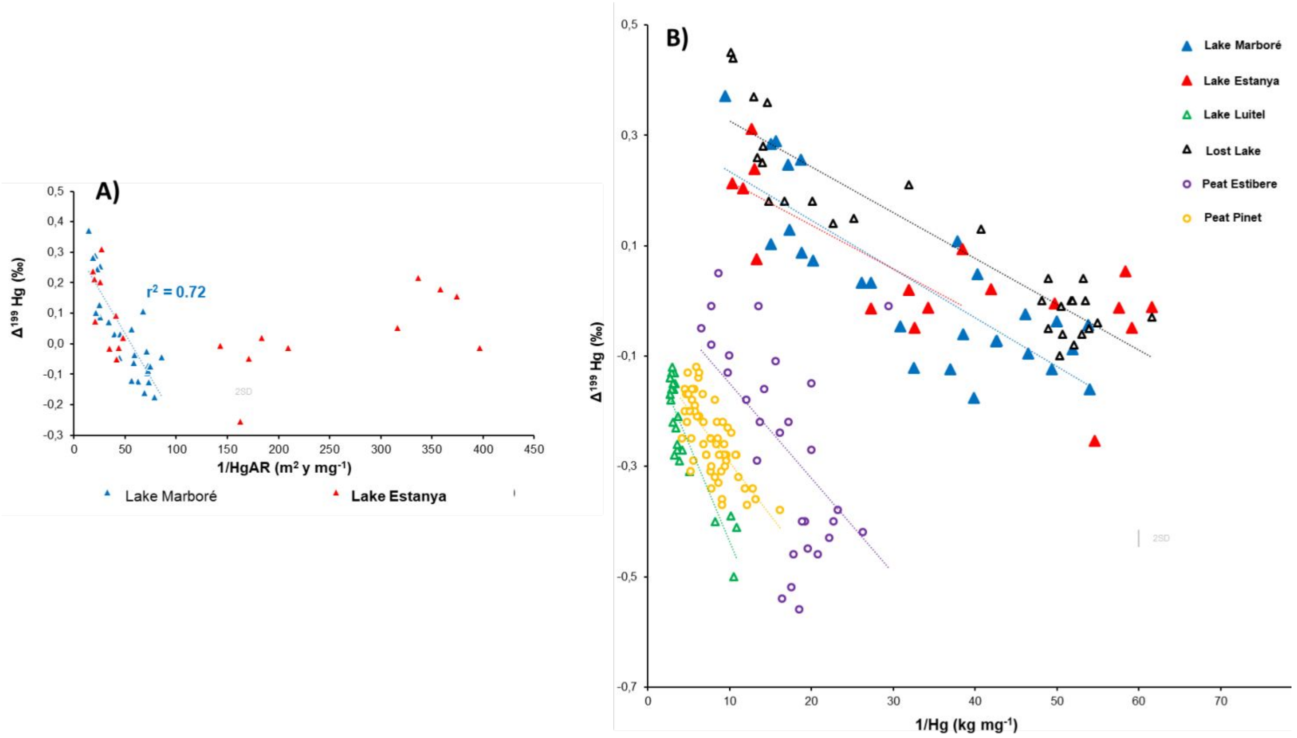

Figure S2. A) Δ^{199}Hg vs 1/HgAR plot for both Lake Marboré and Lake Estanya with strong linear relationship for lake Marboré; B) Δ^{199}Hg vs 1/Hg plot for both Lake Marboré and Lake Estanya, together with other lakes and peats mentioned in the text [4,9-11] to highlight some strong linear relationship (dashed lines)

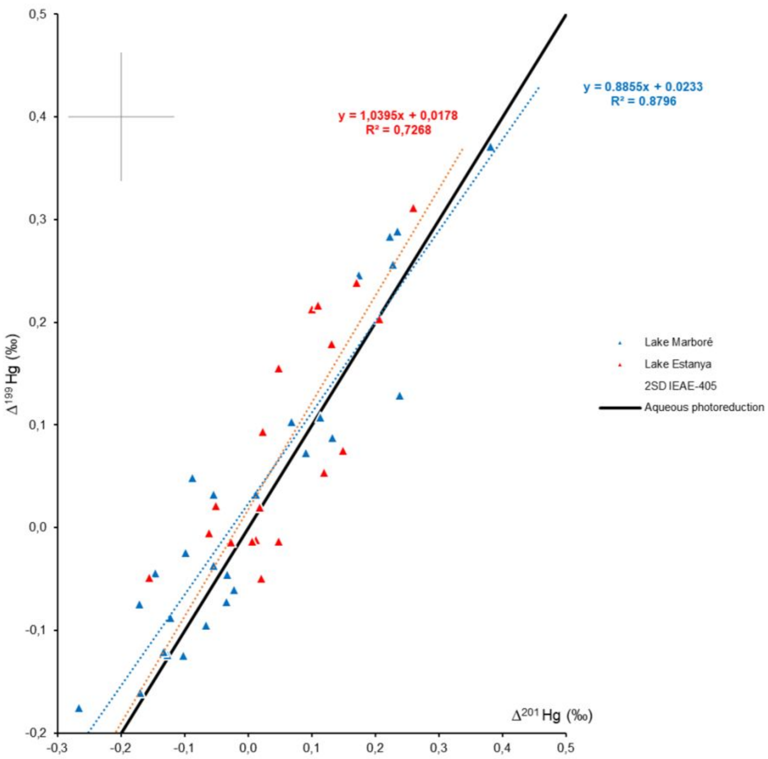

Figure S3. A) Δ^{199}Hg vs Δ^{201}Hg plot for both Lake Marboré and Lake Estanya

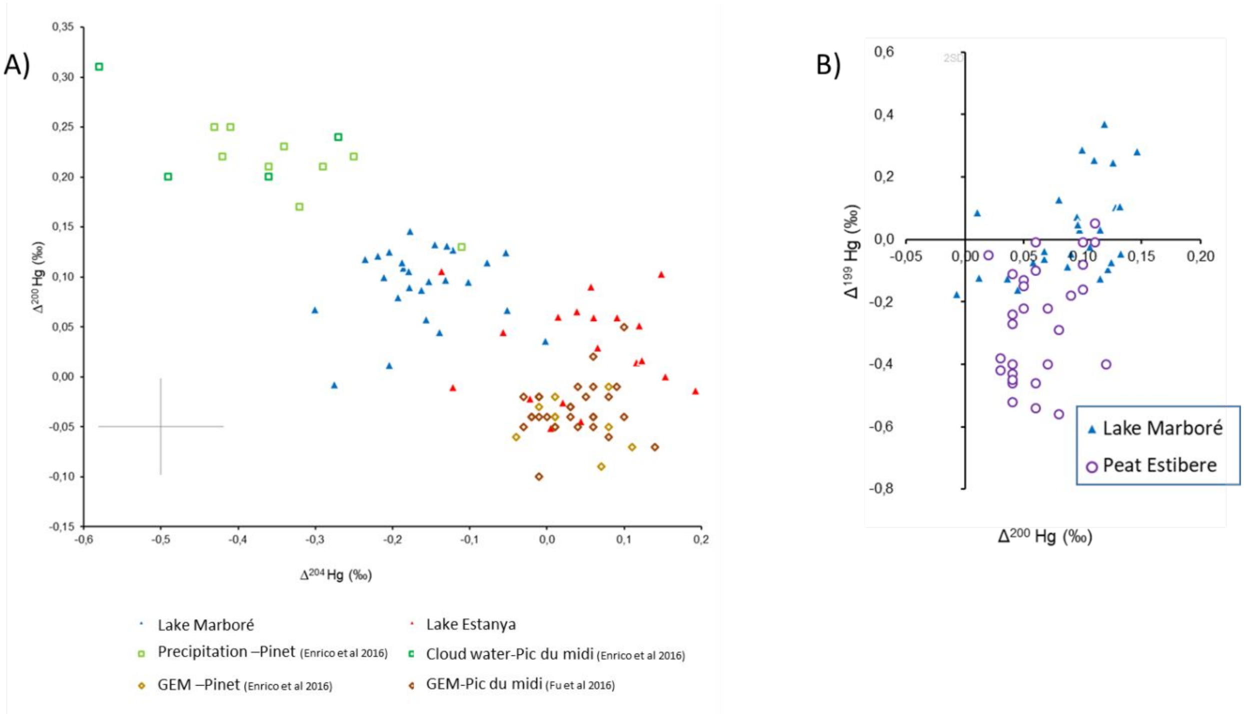

Figure S4. A) Δ^{200}Hg vs Δ^{204}Hg plot for both Lake Marboré and Lake Estanya (this work) and other natural archives and cloud water records mentioned in the text [11,12]; B) Δ^{199}Hg vs Δ^{200}Hg plot for both Lake Marboré and Peat Estibere [10,11]

References

1 Corella, J. P. *et al.* Recent and historical pollution legacy in high altitude Lake Marboré (Central Pyrenees): A record of mining and smelting since pre-Roman times in the Iberian Peninsula. *Science of The Total Environment* **751**, 141557, doi:https://doi.org/10.1016/j.scitotenv.2020.141557 (2021).

2 Blum, J. D. & Bergquist, B. A. Reporting of variations in the natural isotopic composition of mercury. *Analytical and bioanalytical chemistry* **388**, 353-359 (2007).

3 Jiménez-Moreno, M. *et al.* Sources and fate of mercury pollution in Almadén mining district (Spain): Evidences from mercury isotopic compositions in sediments and lichens. *Chemosphere* **147**, 430-438 (2016).

4 Guédron, S. *et al.* A hundred year record of industrial and urban development in French Alps combining Hg accumulation rates and isotope composition in sediment archives from Lake Luitel. *Chemical Geology* **431**, 10-19 (2016).

5 Sherman, L. S. & Blum, J. D. Mercury stable isotopes in sediments and largemouth bass from Florida lakes, USA. *Science of the Total Environment* **448**, 163-175 (2013).

6 Sonke, J. E. *et al.* Sedimentary mercury stable isotope records of atmospheric and riverine pollution from two major European heavy metal refineries. *Chemical Geology* **279**, 90-100 (2010).

7 Ma, J., Hintelmann, H., Kirk, J. L. & Muir, D. C. Mercury concentrations and mercury isotope composition in lake sediment cores from the vicinity of a metal smelting facility in Flin Flon, Manitoba. *Chemical Geology* **336**, 96-102 (2013).

8 Biswas, A., Blum, J. D., Bergquist, B. A., Keeler, G. J. & Xie, Z. Natural mercury isotope variation in coal deposits and organic soils. *Environmental Science & Technology* **42**, 8303-8309 (2008).

9 Kurz, A. Y., Blum, J. D., Washburn, S. J. & Baskaran, M. Changes in the mercury isotopic composition of sediments from a remote alpine lake in Wyoming, USA. *Science of The Total Environment* **669**, 973-982 (2019).

10 Enrico, M. *et al.* Holocene atmospheric mercury levels reconstructed from peat bog mercury stable isotopes. *Environmental science & technology* **51**, 5899-5906 (2017).

11 Enrico, M. *et al.* Atmospheric mercury transfer to peat bogs dominated by gaseous elemental mercury dry deposition. *Environmental science & technology* **50**, 2405-2412 (2016).

12 Fu, X., Marusczak, N., Wang, X., Gheusi, F. & Sonke, J. E. Isotopic composition of gaseous elemental mercury in the free troposphere of the Pic du Midi Observatory, France. *Environmental science & technology* **50**, 5641-5650 (2016).
